# Ion mobility–mass spectrometry of palytoxin-like compounds produced by *Ostreopsis* cf. *ovata*

**DOI:** 10.1007/s00216-025-06158-7

**Published:** 2025-10-16

**Authors:** Noemí Inmaculada Medina-Pérez, Maria Nuria Peralta-Moreno, Jaime Rubio-Martinez, Leïla Bechtella, Lukasz Polewski, Gergo Peter Szekeres, Elisa Berdalet, Encarnación Moyano, Kevin Pagel

**Affiliations:** 1 Department of Chemical Engineering and Analytical Chemistry, University of Barcelona, 08028 Barcelona, Spain; 2 Department of Marine Biology and Oceanography, Institute of Marine Sciences (ICM-CSIC), 08003 Barcelona, Spain; 3 Department of Materials Science and Physical Chemistry, University of Barcelona, 08028 Barcelona, Spain; 4 Institut de Recerca en Química Teòrica I Computacional (IQTCUB), 08028 Barcelona, Spain; 5 Department of Materials Science and Physical Chemistry, University of Barcelona, 08028 Barcelona, Spain; 6 Department of Molecular Physics, Fritz Haber Institute of the Max Planck Society, Berlin, Germany; 7 Department of Chemistry and Biochemistry, Freie Universität Berlin, Berlin, Germany; 8 Water Research Institute (IdRA), University of Barcelona, 08001 Barcelona, Spain

**Keywords:** Palytoxin analogues, Ovatoxins, Ion mobility, Collision cross section

## Abstract

Palytoxin (PLTX) and its analogues from *Ostreopsis* cf. *ovata* are significant health concerns. They show potent vasoconstrictive properties, often causing seafood poisoning. PLTX analogues have chiral centers, resulting in many isomers, making their separation by liquid chromatography and identification/characterization by mass spectrometry challenging. This study explores for the first time the ion mobility spectrometry (IMS) behavior of these compounds to address these analytical challenges. Drift tube ion mobility spectrometry (DTIMS) and traveling wave ion mobility spectrometry (TWIMS) were used and compared. Additionally, trapped ion mobility spectrometry (TIMS) and molecular dynamics simulation were employed to explain unexpected results. TIMS provided higher resolution, distinguishing isomeric ions generated in the electrospray source by losing water molecules from different toxin sites. Computational studies offered theoretical insights into the ion mobility of triply charged calcium and sodium adduct ions, suggesting a folded conformation. ^DT^CCS_N2_ (collisional cross section using DTIMS and nitrogen as buffer gas) values were obtained for PLTX (standard), ovatoxin-a, and ovatoxin-b from microalgae samples in Sant Andreu de Llavaneres (Barcelona, Spain). These values were comparable (ΔCCSs < 2%) to those measured with TWIMS calibrated using PLTX (standard). The study provides 102 CCS values from DTIMS and TWIMS data for adducts and fragment ions of PLTX analogues, which can be used as reference values in databases for toxin screening in complex samples.

**Graphical Abstract:**

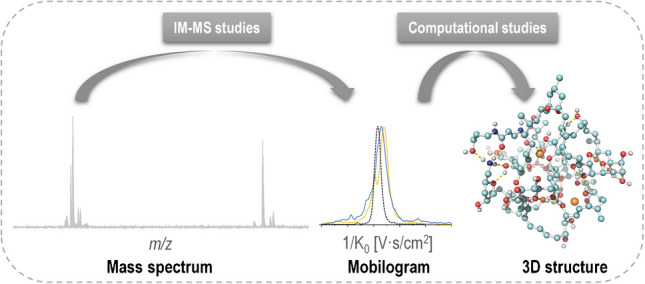

**Supplementary Information:**

The online version contains supplementary material available at 10.1007/s00216-025-06158-7.

## Introduction

Since the late 1990 s, the microalga *Ostreopsis* cf. *ovata* has proliferated on Mediterranean beaches [1–3]. This marine dinoflagellate produces different biotoxins, including isobaric palytoxin and its ovatoxin (OVXT) analogues, which vary depending on sea conditions. Palytoxin (PLTX), recognized as one of the most potent marine biotoxins, has been implicated in severe seafood poisoning in tropical regions and is increasingly concerning along the Mediterranean coast [4].

Currently, PLTX analogues are mainly determined by liquid chromatography-mass spectrometry (LC-MS) methods, which offer high separation and detection capabilities, comprehensive information on the chemical structure, and accurate, selective, and sensitive quantitative analysis. Different OVTX analogues (OVTX-a to OVTX‐i) produced by *Ostreopsis* have been identified through multiple-stage mass spectrometry (MS^n^) and high-resolution mass spectrometry (HRMS) [5, 6]. Additionally, the isobaric palytoxin produced by the microalga has been identified as a structural isomer of palytoxin produced by the coral *Palythoa tuberculosa* [7].

**Fig. 1:**
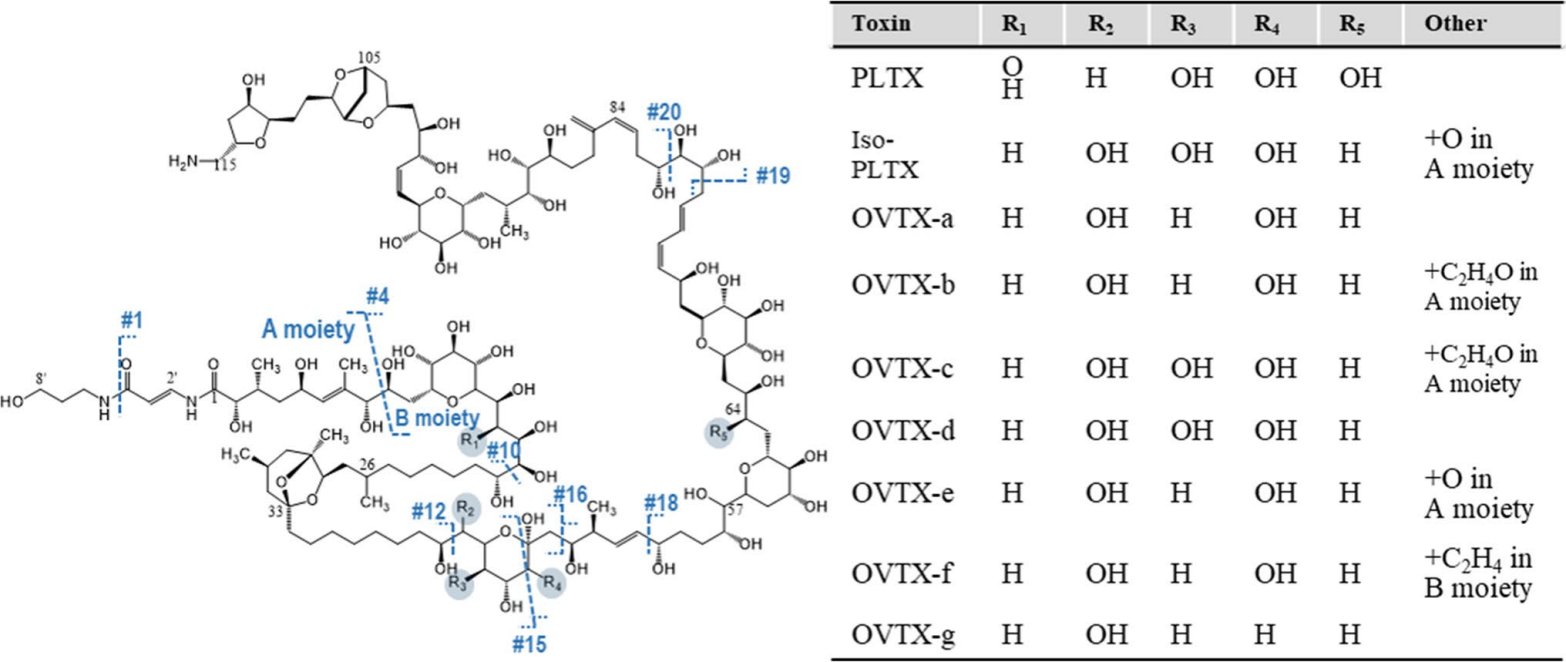
Chemical structures of palytoxin and its analogues (iso-PLTX, OVTX-a to OVTX-g) commonly found in *Ostreopsis* cf. *ovata* samples from the North West Mediterranean. The cleavage numbering follows the general fragmentation pattern proposed by Ciminiello et al. [11]

Over the past few decades, ion mobility-mass spectrometry (IM-MS) has emerged as a powerful analytical technique for the separation of gas-phase ions, enabling differentiation based on size, shape, and charge [8–10]. The integration of ion mobility spectrometry (IMS) with high-resolution mass spectrometry (HRMS) enhances both separation and identification capabilities, allowing for the unequivocal characterization of complex compounds. A key advantage of IMS is its ability to generate collision cross section (CCS) values, which are unique to each ion and correlate with its three-dimensional structure. This capability is particularly valuable for distinguishing structural isomers, isobaric species, and characteristic fragment ions of palytoxin and ovatoxins, thereby providing critical insights into their conformational dynamics and structural complexity. Although PLTX and ovatoxins (OVTXs) share a similar chemical structure backbone, these molecules contain over 60 chiral centers, and their analogues differ in the positioning of the hydroxyl groups (Fig. 1), Such structural diversity poses significant challenges for separation and identification using conventional techniques [11]. These differences can markedly influence gas-phase conformations and, consequently, their ion mobility profiles. IMS is uniquely suited to detect these subtle structural variations, as shifts in hydroxyl group location and modifications in molecule conformation can alter the collision cross section (CCS) values, enabling differentiation of analogues based on their drift times. This project represents the first application of IM-MS to enhance isomer/isobaric separation and gain deeper insights into the conformation of palytoxin and ovatoxins.

The structural diversity of palytoxins and their analogues, such as ovatoxins, plays a critical role in their toxicological behavior. Even minor variations in stereochemistry or functional groups can significantly alter their interactions with biological membranes and ion channels, leading to differences in cytotoxicity and systemic effects. For instance, ovatoxin-a has been shown to be less toxic than palytoxin itself, while ovatoxin-d exhibits even lower toxicity, particularly in models assessing intestinal barrier integrity. Moreover, recent studies have identified isobaric forms of palytoxin with distinct fragmentation patterns and potential toxicological implications. These findings underscore the importance of characterizing each isomer and analogue individually to better assess the environmental and health risks associated with *Ostreopsis* blooms [6, 12].

In this context, the use of ion mobility mass spectrometry (IM-MS) provides a powerful tool to resolve and study the conformational landscape of these complex molecules. The identification of potential conformers is not only analytically novel but also biologically relevant, as different conformers may exhibit distinct toxicological profiles due to variations in their three-dimensional structures and binding affinities. Understanding these conformational differences is essential for accurate risk assessment, regulatory monitoring, and the development of structure–activity relationship (SAR) models. Therefore, the ability to distinguish and characterize individual conformers contributes directly to improving public health responses and environmental safety strategies related to harmful algal blooms. Nowadays, several technologies are available for ion separation via ion mobility spectrometry (IMS). The most commonly used technologies to combine IMS with LC-HRMS are Drift Tube Ion Mobility Spectrometry (DTIMS), Traveling Wave Ion Mobility Spectrometry (TWIMS), and Trapped Ion Mobility Spectrometry (TIMS). DTIMS systems separate ions in a drift tube pressurized with a drift gas (typically N_2_ or He) and apply a uniform electric field to propel the ions toward the mass analyzer region. As ions traverse the ion mobility cell, they are slowed down by the inert drift gas based on their size, charge, and shape. Furthermore, the reduced ion mobility constant (K_o_), which can be derived from the observed drift time, allows for the calculation of the corresponding collision cross section (CCS) value (a molecular property independent of instrumental conditions). This value is useful for characterizing the chemical structure of ions. In DTIMS, a primary calibration method is used to calculate ^DT^CCS, which serves as a reference to calibrate other ion mobility techniques such as TWIMS and TIMS. These techniques require secondary calibration methods to ensure reliable and comparable CCS values. In TWIMS, a non-uniform electric field generates a sequence of symmetric potential waves that continuously propagate through the ion mobility cell, driving the ions towards the mass analyzer. The ions “surf” on the waves and reach the mass analyzer based on their *K*_*o*_. Conversely, in the TIMS instrument, ions are propelled by a gas flow to traverse the mobility cell towards the mass analyzer while an electrical field is applied in the opposite direction, trapping and accumulating the ions in the first region of the cell. In the second part of the mobility cell, ions are released by progressively decreasing the electric field and separated according to their reversed reduced ion mobility constant (1/K_o_).

This work aims to study PLTX and OVTXs produced by *Ostreopsis* for the first time using IM-MS with both DTIMS and TWIMS, and to calculate the CCS values of ions observed in electrospray. Additionally, to achieve a deeper understanding of these analytes, the higher ion mobility resolution of TIMS and data from computational studies of molecular dynamics simulation are also applied.

## Material and methods

### Reagents and solvents

Palytoxin standard (PLTX_std_) (from *Palythoa tuberculosa*) was obtained from Wako Chemicals GmbH (Germany). A stock standard solution (100 mg/kg) was prepared by weight in MeOH:H_2_O (50:50, *v/v*). PLTX_std_ working solutions were prepared by diluting the stock standard solution in MeOH:H_2_O (80:20, *v/v*). All standard solutions were stored at − 20 °C until use. Water, acetonitrile, formic acid, and methanol (LC–MS grade > 98%) used for preparing mobile phases and standard solutions were purchased from Sigma-Aldrich (St. Louis, USA).

Dextran from Leuconostoc mesenteroides Mw 1000, used as a calibrant in TWIMS experiments, was purchased from Sigma-Aldrich (St. Louis, USA). The calibrant solution was prepared by dissolving the Dextran Mw 1000 to a concentration of 0.5 µg/µL in H_2_O:MeOH (1:1, *v/v*).

### Samples

Samples extracts containing OVTXs were obtained from *Ostreopsis* cf. *ovata* samples collected during the 2021 bloom. The most concentrated samples, collected from 28th July to 2nd August, were selected for this study. These samples were collected and extracted following the previously described method [13, 14]. Briefly, 5 to 20 g of the dominant macroalgae was carefully collected and transferred into a 250 mL plastic bottle containing approximately 180 mL of in situ sea water. These samples were vigorously shaken and sieved through a 200 µm mesh, and the percolated water was filtered through duplicate GF/F grade glass fiber filters. These filters were extracted with 2 mL of MeOH:H_2_O (80:20, *v/v*) for 5 min in an ice-cooled ultrasonic bath, followed by vortexing and centrifugation. The supernatant was filtered through 0.22 µm nylon membranes and stored at −  80 °C until fractionation. Finally, the OVTXs extracts were fractionated by LC-UV into individual toxin fractions. The collected fractions were stored in amber glass vials at − 80 °C until analysis by IM-MS.

### Instrumentation and analytical procedure

#### Liquid chromatography with UV detection

The reversed-phase chromatographic separation was conducted on a Hypersil GOLD C18 column (100 mm × 2.1 mm i.d., 1.9 µm particle size) packed with totally porous silica particles (Hypersil, Thermo Fisher Scientific). The separation was performed under gradient elution mode using acetonitrile as solvent A and water as solvent B, both containing 0.1% formic acid. The gradient elution program began with 30% solvent A for 1.5 min, followed by a linear increase to 35% solvent A over 12 min. Subsequently, solvent A was increased to 90% for 1.5 min, and these conditions were maintained in an isocratic step for 1 min before returning to the initial conditions. The mobile phase flow rate was 300 µL/min, with the column temperature held at 23 °C, and 20 µL of each sample as injection volume. OVTX-like compounds were detected by monitoring absorbance at their characteristic wavelengths, 233 and 263 nm.

#### Nano-electrospray ionization (nESI)

Direct infusion analyses were performed in positive ion mode using platinum/palladium (Pt/Pd, 80/20)-coated borosilicate capillary emitters prepared in-house. Typically, 5–10 μL of the samples were loaded on nESI emitters and ionized by applying voltages in the range of 1.0–1.5 kV.

The source temperature was maintained at 150 °C, with a cone gas flow rate of 1 L/h. The sampling cone voltage was set at 30 V, and the extraction cone voltage was 25 V when using Synapt G2-S (DTIM and TWIM) instruments (Waters, UK).

A home-made off-line nESI source [15] was employed for direct infusion with the timsTOF (Bruker, Germany). The applied conditions included a drying gas flow rate of 3 L/min at 150 °C, an end plate offset of 500 V, and a nebulizer pressure of 0.1 bar.

#### Drift tube ion mobility-mass spectrometry (DTIM-MS)

Drift tube IM-MS measurements were performed using a modified Synapt G2-S HDMS instrument (Waters, Manchester, UK), as detailed by Bush et al*.* and others [16–18]. In this modified Synapt, the traveling wave ion mobility cell was replaced with an rf-confining drift tube, enabling the determination of ^DT^CCSs values using helium and nitrogen as buffer gases (1.8 Torr). The ion mobility parameters were as follows: cell DC voltages were 60 V with He as drift gas and 100 V with N_2_; drift voltage range from 40 to 240 V; gas flow rates were 2 mL/min in the trap and 90 mL/min in the IM cell. To study the ion mobility of product ions, tandem MS experiments were performed prior to ion mobility separation using N_2_ as collision gas, with collision energies ranging from 25 to 50 eV. Mass spectra were acquired from *m/z* 100 to 1500. Table S1 summarizes the observed product ions, along with the corresponding precursor ions and collision energies used in both DTIM-MS and TWIM-MS instruments. Mass Lynx v4.1 (Waters) software was used to control the instrument (DTIMS) and to acquire and process the LC-IM-HRMS data.

#### Traveling wave ion mobility-mass spectrometry (TWIM-MS)

Traveling wave IM-MS measurements were conducted on a Synapt G2-S HDMS instrument (Waters, Manchester, UK) using N_2_ as a buffer gas. The settings were as follows: 45.0 V trap DC bias voltage, 2 mL/min trap gas flow, 90 mL/min cell gas flow, 650 m/s IM wave velocity, 40.0 V IM wave height, 20 mL/min IM gas flow, 380 m/s transfer wave velocity, and 5.0 V transfer wave height voltage. In tandem MS experiments, argon was used as a collision gas with collision energies ranging from 32 to 60 eV. Mass spectra data were acquired within the *m/z* range of 100–1500. Mass Lynx v4.1 (Waters) software was also used to control the instrument (TWIM-MS) and to acquire and process the LC-IM-HRMS data.

#### Trapped ion mobility spectrometry-mass spectrometry (TIMS-MS)

Measurements using the trapped ion mobility-mass spectrometry (TIMS-MS) instrument were performed on a timsTOF Pro from Bruker (Bremen, Germany). The reversed ion mobility (1/K_0_) range was from 0.6 to 1.6 V s/cm^2^, with N_2_ as the drift gas. RF amplitudes for Funnel 1 and Funnel 2 were set to 300 Vpp and 400 Vpp, respectively. The transfer hexapole operated at a deflection delta voltage of 30 V and an RF amplitude of 400 Vpp. The quadrupole ion energy was 3 eV, with the “low mass” set to *m/z* 300. Transfer time and pre-pulse storage time were 70 µs and 15 µs, respectively. The mass range was set to *m/z* 100–3000 and the collision energy for tandem MS experiments ranged from 20 to 65 eV. Bruker Compass Analysis Viewer Software 5.3 was used to control the instrument and to acquire and process the LC-IM-HRMS data.

#### Collisional cross section (CCS) measurements

CCS values were calculated from ion mobility drift times. ^DT^CCS_He_ and ^DT^CCS_N2_ values were determined using the stepped field method by plotting the arrival times (centroid of the best-fit Gaussian) as a function of reciprocal drift voltages and measured using the Mason-Shamp equation [19]. The PLTX standard (PLTX_std_) (1 µg/mL), OVTX-a, and OVTX-b were analyzed using ten different drift voltages that increase axial potential across the drift tube. The drift voltage range was 40–240 V with He and 100–240 V with N_2_ as buffer gases, while the gas pressure was always 1.8 Torr. Ion mobility data were acquired for 60 s at each electric field. Compounds were injected in triplicate on the same day (intra-day) and over three consecutive days (inter-day) to calculate the ^DT^CCS repeatability using both He and N_2_ as buffer gases. Data were processed with Mass Lynx v4.1 software (Waters). The drift times were extracted, and CCS values were calculated using in-house developed software. Details about the code and modules can be found online (https://github.com/jeriedel/CCS).

The TWIMS system employed secondary calibration methods using ^DT^CCS values previously measured for a selected calibrant. This approach corrected the relationship between traveling wave drift time and mobility values for mass dependence, yielding corrected drift time (dt′) and corrected CCS values (CCS′) [20, 21]. The linear correlation provided the adjusted parameters of the equation (*CCS')* = *A(dt')* + *N*, from which the estimated CCS values for PLTX analogues were obtained by interpolating the corresponding dt′ and CCS′. The TWIMS calibration was performed using two calibrants: dextran oligosaccharides and PLTX_std_. Dextran oligosaccharides were used due to their well-established CCS values and suitability for covering a broad mass and mobility range. PLTX_std_ was included as a secondary calibrant to improve accuracy in the high mass range, where dextran coverage is limited. Importantly, multiple ions derived from PLTX_std,_ whose CCS values had been previously determined using DTIMS, were used in the calibration process. This allowed us to perform a multi-point calibration rather than relying on a single reference value, ensuring robustness and reliability in the CCS measurements for palytoxin-related compounds. Both calibrants were injected in triplicate on the same day (intra-day) to calculate the ^TW^CCS repeatability using N_2_ as a buffer gas. Data were processed with Mass Lynx v4.1 software (Waters) and OriginPro 8.5 (OriginLab, Northampton, UK).

### Computational study details

#### System preparation

Starting from the simplified molecular-input line-entry system (SMILES) ASCII string of PLTX_std_, an initial guess of the 3D structure of the molecule was generated using the Online SMILES Translator and Structure File Generator from the NIH (https://cactus.nci.nih.gov/translate/). The system was then prepared using Maestro 2016−2 software [22]. Parameters and partial charges were obtained using Antechamber from AMBER20 [23] employing the AM1-BCC model [24]. To represent and explore the conformational space of the toxin, 15 different initial random positions of Ca^2+^ and 2Na^+^ were considered in the simulation of each of the [M+H+Ca]^3+^ and [M+H+2Na]^3+^ systems. The same protocol was applied to OVTX-a and OVTX-b.

#### Energy minimization and Molecular Dynamics Simulation

Prior to any molecular dynamics (MD) simulation, the chemical structure was initially relaxed using a 10,000-step minimization protocol with the steepest descent approach. Gas-phase conventional Molecular Dynamics (cMD) simulations of 1 µs duration were performed in a vacuum (igb = 6, cutoff = 999.99) using AMBER20 in its GPU CUDA version [25] for all adducts. The generalized Amber forcefield (GAFF2) and the ff14SB.water force field were employed for the simulations [26].

#### Analysis of computational results

To analyse the trajectories, the Cpptraj AMBER20 module was employed [27]. Structural stability of the system was evaluated using Root-Mean Square Deviation (RMSD) analysis. To examine the structural conformations explored, hierarchical clustering was performed using the average linkage algorithm. Additionally, the conformational diversity of the studied adducts was analyzed by inspecting the radius of gyration (Rg). Donor-acceptor hydrogen bond interactions established during the trajectory were also evaluated.

## Results and discussion

**Fig. 2:**
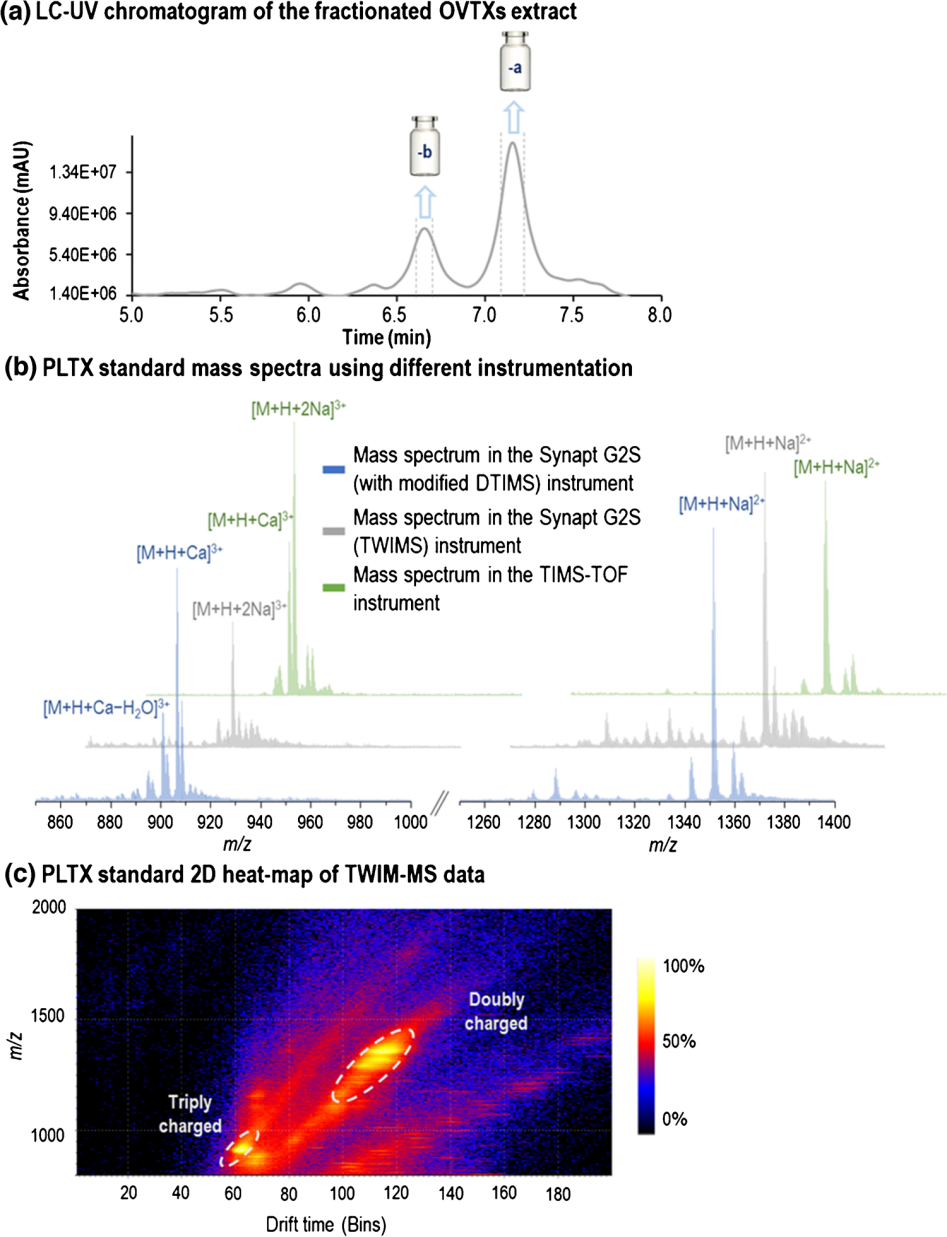
(**a**) LC-UV chromatogram of the fractionated sample, (**b**) mass spectrum of the palytoxin standard obtained using various instrumentations, and (**c**) 2D heat-map of TWIM-MS data for the PLTX_std_

To study the ion mobility behavior of PLTX analogues, sample extracts were separated by UHPLC to collect fractions enriched with these compounds. Figure 2a shows the UHPLC-UV chromatogram of the sample extract, indicating the retention time windows for the collected fractions (A and B). Fractions collected from multiple injections were pooled and used for further nESI-IM-HRMS analysis with three ion mobility instruments. The pooled fractions A and B contained OVTX-a and OVTX-b, respectively. The MS profile observed for each toxin included adduct ions (with calcium, sodium, magnesium, and potassium), as well as fragment ions formed by multiple water losses due to in-source fragmentation. The ions observed are listed in Table S2. As shown in Fig. 2b, the most abundant ions were triply charged ions, [M+H+Ca]^3+^ and [M+H+2Na]^3+^, and doubly charged ions, [M+H+Na]^2+^. It should be noted that not all ions observed in the DTIMS system were also observed in the TWIMS and TIMS instruments. This aligns with our previous findings, which indicated that the nature, intensity, and relative abundance of ions generated in the electrospray source by these toxins may vary depending on the instrumentation used, their maintenance, and working conditions.^13^ Furthermore, there is no evidence for the potential presence of isomers or isobaric ions that coelute with diagnostic ions in LC. The combination of nESI-HRMS and ion mobility for the analysis of PLTX analogues provides an additional dimension that helps to better characterize these toxins and natural extracts. CCS values have also been calculated for all the ions observed in DTIMS and TWIMS instruments.

As an example, Fig. 2c shows the heat-map visualization of a representative mobilogram obtained with TWIMS, where the doubly and triply charged ions of PLTX_std_ generated in nESI can be easily distinguished. Furthermore, the higher ion mobility resolution provided by TIMS was useful for separating and studying possible isomers produced by the loss of multiple water molecules.

**Fig. 3:**
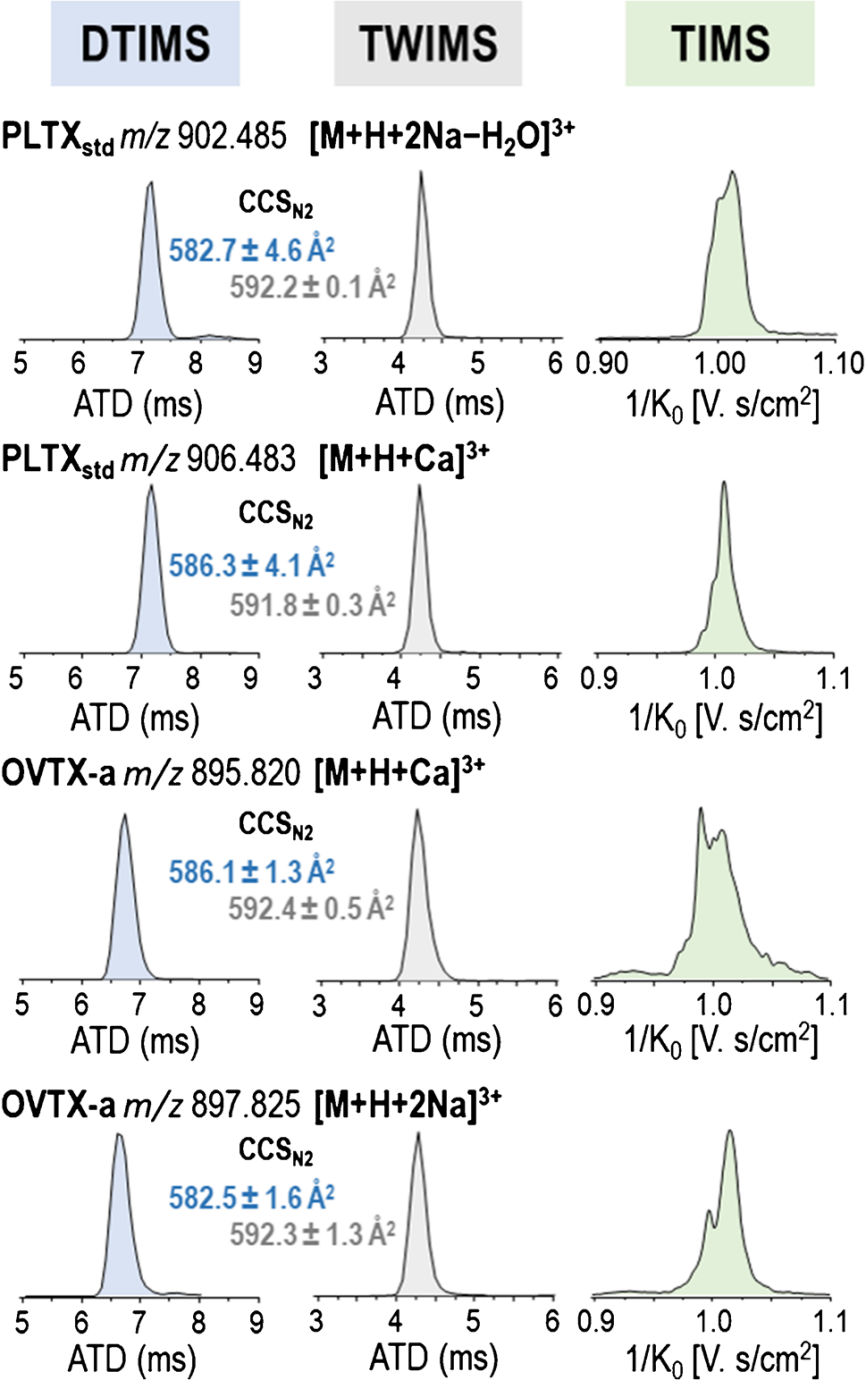
Comparison of ion mobility peaks observed in DTIMS, TWIMS, and TIMS for selected adduct ions and toxins. CCS values, determined on three replicates, along with their corresponding standard deviations, are indicated for DTIMS and TWIMS measurements

**Fig. 4:**
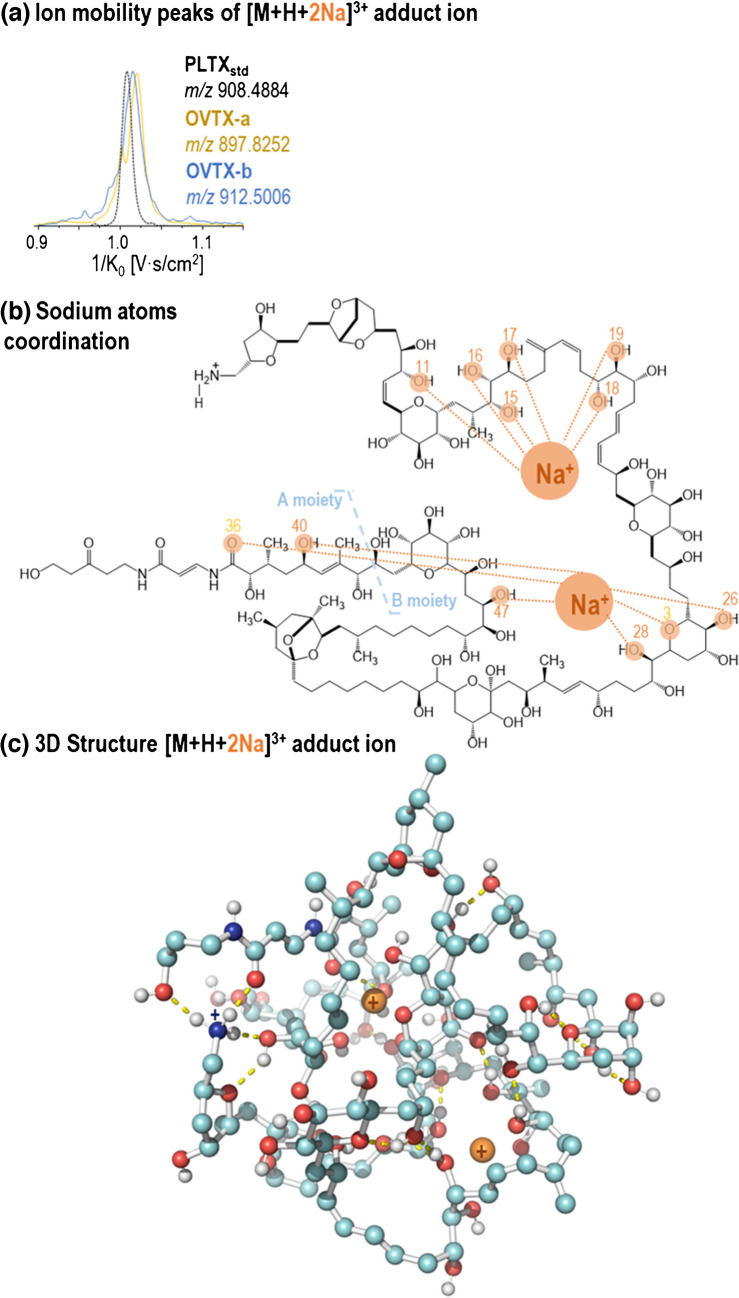
(**a**) Ion mobility peaks of the [M+H+2Na]^3+^ adduct ion by TIMS for the three toxins. As an example, (**b**) coordination (dashed lines) of Na ion for the OVTX-b structure, and (**c**) 3D structure of the [M+H+2Na]^3+^ adduct ion corresponding to OVTX-b. Yellow dashed lines indicate the hydrogen bonds. Details of the hydrogen bonds can be found in Table S3 and Fig. S2

**Fig. 5:**
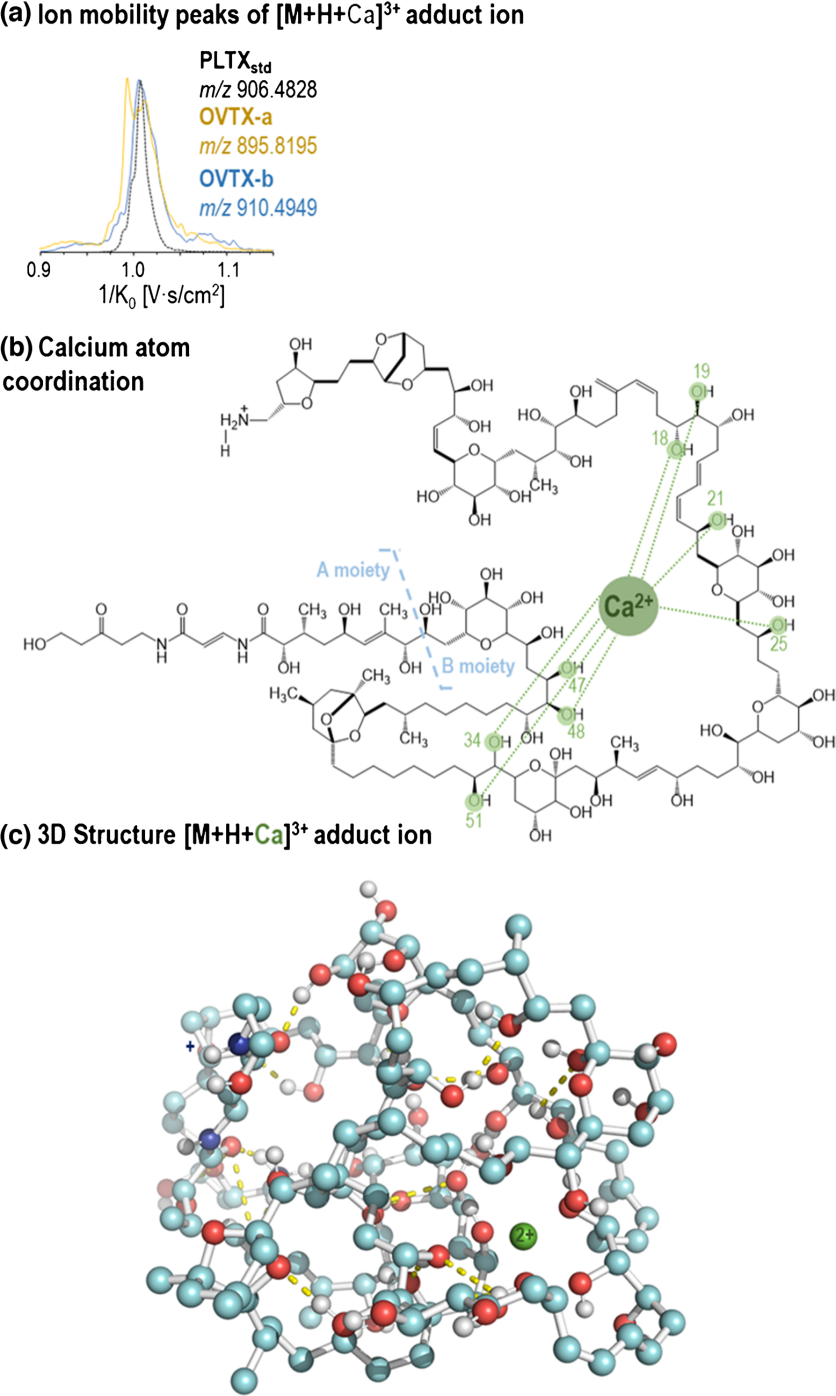
(**a**) Ion mobility peaks of the [M+H+Ca]^3+^ adduct ion by TIMS for the three toxins. As an example, (**b**) coordination (dashed lines) of Ca ion for OVTX-b, and (**c**) 3D structure of the [M+H+Ca]^3+^ adduct ion corresponding to OVTX-b. Yellow dashed lines indicate hydrogen bonds. Details of the hydrogen bonds can be found in Table S3 and Fig. S2

Figure 3 shows selected ion mobility peaks corresponding to triply charged ions of PLTX_std_ and OVTX-a (among the most abundant ions) observed using the three IMS instruments (DTIMS, TWIMS, and TIMS). For the selected ions, only one peak was observed in both DTIMS and TWIMS, while more than one mobility peak or a distorted peak was observed in TIMS, likely due to the overlap of multiple structures (isomers). Despite the differences in the chemical structure and molecular weight of these triply charged ions, similar CCS values were obtained (Fig. 3, details in Table S2), indicating that these toxin ions are of comparable size. The larger number of peaks and broader peaks observed in TIMS could be related to the different sites where each metal cation interacts to form the corresponding adduct ion or to the different sites where water molecules were lost, resulting in various conformational structures. As shown in Fig. 4a and Fig. 5a, the TIMS results indicated that the Na/Ca-triply charged adduct ions, [M+H+2Na]^3+^ and [M+H+Ca]^3+^, had values within a very narrow window of 1/K_0_, regardless of the toxin analogue.

To explain these observations, computational analysis was performed using molecular dynamics simulations for PLTX and OVTXs (-a and -b). Due to the high degrees of freedom (torsions, dihedral angles, etc.) presented by the structures, the minimization process carried out prior to any simulation indicated that these molecules tend to fold. Initial visual inspection of the dynamic simulations revealed folding structures’ stabilization by electrostatic interactions and hydrogen bonds. Furthermore, simulations with 2Na^+^ and Ca^2+^ cations indicated that these cations could form adduct ions through coordination numbers of 6 and 8, respectively (Fig. 4b and Fig. 5b), facilitated by interactions with oxygens from different functional groups. Comparing the dynamic results, no clear preference for specific binding sites was observed. However, the interaction is maintained throughout the trajectory of the cations within the chemical structure, favored by the “folded and compacted” structure that brings certain regions of the toxin closer together. For example, Fig. S1 provides additional perspectives on the tridimensional clustered structures (most prone) of the different adduct ions, where the 2Na (orange) and the Ca (green) cations are coordinated. Figure 4c and Fig. 5c illustrate the hydrogen bonds (yellow dashed lines) that could stabilize the “folded and compacted” structure of these toxins. Figure S2 and Table S3 also summarize the specific hydrogen bonds that are more likely to stabilize the structure of each adduct ion.

Although the simulations revealed different possible conformations, the volume of the molecule appeared to be similar, with no significant differences observed between the radius of gyration of the adduct ions, [M+H+2Na]^3+^ and [M+H+Ca]^3+^ (Fig. S3). It can be suggested that these cations tend to stabilize the "folded" structure by coordination within the "inner" sites. Despite possible conformational interconversions, the radius of gyration remains stable with slight variations for each ion throughout the dynamics. However, the averaged measurements indicate that it is slightly higher in the case of the calcium adduct (Fig. S3), which aligns with the calculated ^DT^CCS_N2_ values (^DT^CCS_N2_ [M+H+Ca]^3+^  = 586 ± 1 Å^2^, ^DT^CCS_N2_ [M+H+2Na]^3+^  = 583 ± 2 Å^2^). In this context, it can be hypothesized that the distorted peaks of these adduct ions observed in TIMS (Fig. 3) are due to the different conformations that this structure could adopt in the gas phase, leading to slight variations in the radius of gyration observed, while cations could be moving within the inner sites of the molecule.

**Fig. 6:**
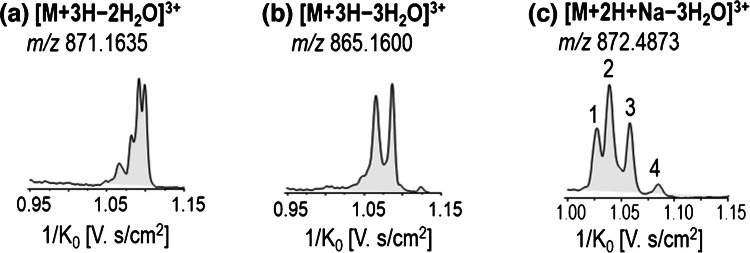
Triply charged ions observed with water losses for OVTX-a using TIMS: (**a**) [M+3H−2H_2_O]^3+^; (**b**) [M+3H−3H_2_O]^3+^; (**c**) [M+2H+Na−3H_2_O]^3+^

In addition, the loss of water molecules could occur from several hydroxyl groups, resulting in different isomers. If the hydroxyl groups involved in water losses are located at the peripheral sites of the folded molecule (as indicated in Fig. S4), different arrival times (ion mobility values) would be expected for the isomers generated in this process (Fig. 6). This phenomenon was only observed when working with higher ion mobility resolution in TIMS, as the loss of water molecules could significantly affect the volume of these ions and consequently their ion mobility. However, their signal intensity was insufficient for reliable CCS determination in DTIMS and TWIMS. As a result, these ions were not included in Tables S1 and S2. Further computational analysis will be carried out in future studies to more accurately explain the observations in the TIMS instrument.

Moreover, the isomer ions observed under the same *m/z* but with multiple reversed reduced ion mobilities (1/*K*_*o*_) were fragmented individually in the TIMS-TOF instrument by applying high energy in the collision cell before the high-resolution mass measurement. For example, Fig. S5 shows the mass spectra of fragment ions observed for the ion [M+2H+Na−3H_2_O]^3+^ of OVTX-a (Fig. 6c). The same fragmentation pattern was observed for the different isomers, regardless of the sites where the water loss occurred. Furthermore, peak #4, corresponding to the isomer with the highest reversed reduced mobility (1/*K*_*0*_ = 1.084 V s/cm^2^)—and thus the lowest reduced mobility *K*_*0*_—is likely the most voluminous and structurally expanded ion, closely resembling the unfragmented toxin ion prior to water loss. These results suggest that in this ion, the water loss could occur at the inner site of the molecule without affecting the final volume. In contrast, the isomer #1, which shows the lowest reversed reduced mobility (1/*K*_*0*_ = 1.027 V s/cm^2^) (the highest *K*_*0*_), could be associated with water losses occurring at the outer part of the toxin structure, resulting in a more compact isomer. Additionally, the fragment ion at *m/z* 327.1914 can be assigned to [M+H−B moiety−H_2_O]^3+^, indicating that one of the water losses could occur in the A moiety (Fig. 1). The fragment ion at *m/z* 1136.1299 can be assigned to [M+H+Na−A moiety−3H_2_O]^2+^, indicating that one Na^+^ conjugation occurs within the B moiety of the molecule (Fig. 4b) and the three simultaneous water losses occur in this B moiety. These observations are consistent with the dynamic simulation results. *Determination of CCS values.*

In the last decade, CCS values have been increasingly utilized, in combination with retention time, exact mass, and tandem MS data, to identify and characterize molecules in complex matrices and to enhance the selectivity and sensitivity of analytical methods. Currently, there is no information on ion mobility and CCS values for PLTX and OVTXs. Including this structural parameter in the databases is therefore of great interest to aid in the screening of these toxins. In this work, for the first time, CCS values have been calculated for the different ions observed in nESI-IM-HRMS for PLT_std_, OVXT-a, and OVTX-b. Table S2 summarizes CCS values calculated for 102 ions observed in nESI and measured in both DTIMS and TWIMS systems, using two drift gases (He and N_2_). The ^DT^CCS and ^TW^CCS values were measured in triplicates on three different days, providing intra- and inter-day precision with an RSD% lower than 2%. Based on these results and current literature, a deviation within ±2% between experimental and reference CCS values is generally considered an acceptable threshold for the reliable identification of unknown species using IMS-MS [28]. Comparison of the results using He and N_2_ as buffer gas showed that ^DT^CCS_N2_ values were higher than ^DT^CCS_He_ values (Table S2), in agreement with previous studies on other compounds [16, 29, 30]. These observations can be attributed to the higher (>eightfold) polarizability of N_2_ (N_2_: 1.74 × 10^−24^ cm^3^, He: 0.21 × 10^−24^ cm^3^) [19, 31], which may undergo more significant distortion by the electric field during ion-neutral collisions, resulting in larger changes in the shape or conformation of the ions. Differences between ^DT^CCS_He_ and ^DT^CCS_N2_ for doubly and triply charged ions ranged from 12 to 14%, but these differences increased to 16–21% for smaller ions such as singly charged fragment ions.

Since ^DT^CCS_N2_ values are widely accepted as reference standards, those calculated for PLTX_std_ ions were tested as calibrants for determining TWIMS CCS (^TW^CCS_N2_) and compared with a calibration method based on a dextran ladder. Table S2 summarizes the ^TW^CCS_N2_ values calculated for ions observed in PLTX analogues using both dextran and PLTX_std_ as calibrants. Figure S6 illustrates the correlation between ^DT^CCS_N2_ and ^TW^CCS_N2_ values using dextran and PLTX_std_ as calibrants. A higher determination coefficient (R^2^ = 0.996) and a slope closer to 1 (slope = 1.02) were obtained when using PLTX_std_ as the calibrant, indicating it provides more reliable ^TW^CCS_N2_ values for PLTX analogues. The differences between ^DT^CCS_N2_ and ^TW^CCS_N2_ values were within 0.9 ± 0.6% when using PLTX_std_, compared to higher differences of 4.6 ± 1.6% when using dextran. This discrepancy can be attributed to several factors, like ion heating during transfer, the specific calibration model used, and concentration-dependent Coulombic effects. In this study, the use of multiple ions generated from PLTX_std_ was used to calibrate the TWIMS system during the analysis of PLTX analogues. This allowed for a more representative calibration across the relevant mass and mobility range and enabled re-calibration procedures to correct for deviations. These observations support the conclusion that selecting a calibrant with a chemical nature similar to that of the analytes yields more accurate and reliable CCS estimations.

**Fig. 7:**
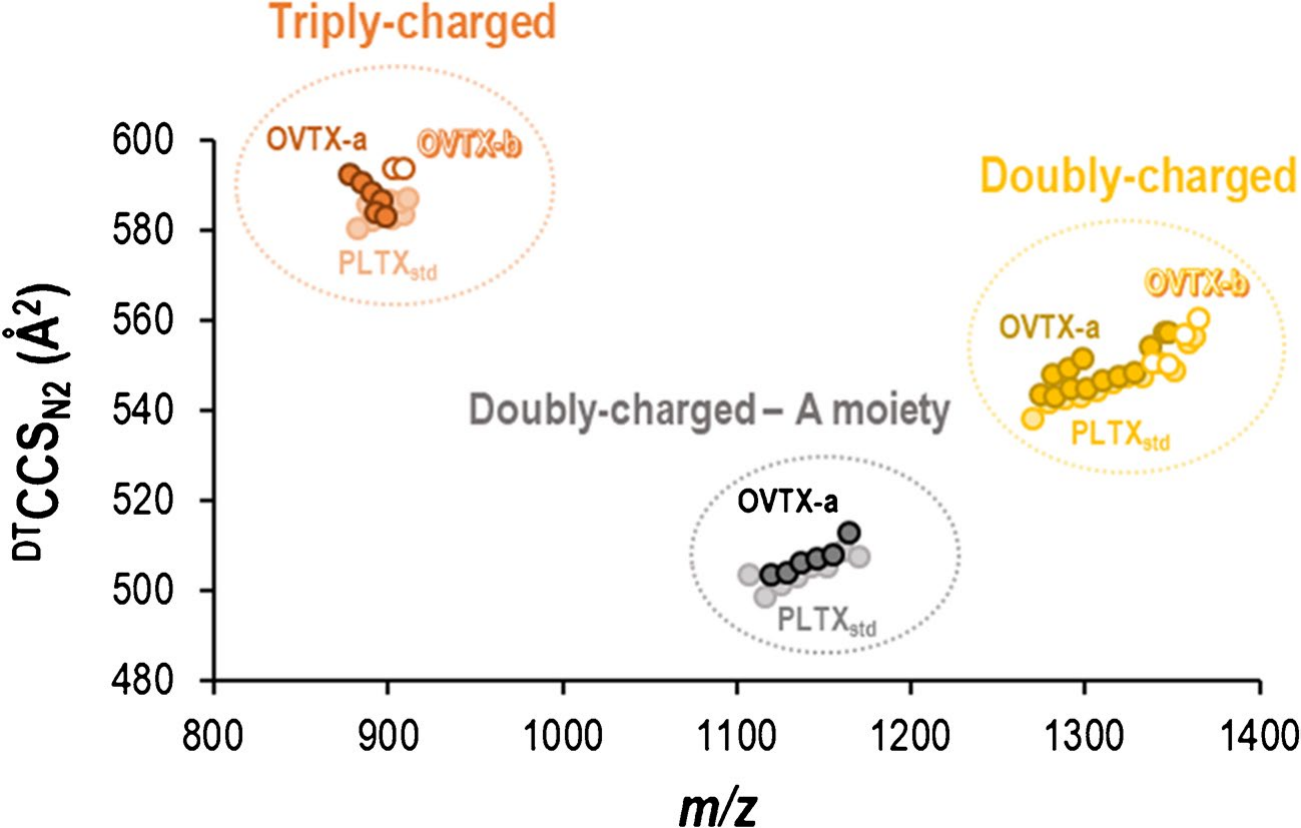
Conformational space map of the stepped field absolute ^DT^CCS_N2_ values for the doubly and triply charged ions (adducts) of toxins PLTX_std_, OVTX-a and OVTX-b, as well as the doubly charged A moiety fragment of toxins PLTX_std_ and OVTX-a detected using DTIMS in the 800–1400 *m/z* range

Figure 7 presents the CCSs values measured for different toxins versus their corresponding *m/z* values. The ^DT^CCS_N2_ values range between 497 and 593 Å^2^. For the different PLTX analogues with the same charge state, the CCS values fall within a narrow range, suggesting that minor structural differences do not significantly affect the spatial conformation of the ions. The CCS value of the same type of ion across different toxins (Table S2) differed by less than 1.1%. Additionally, doubly and triply charged toxins are distinctly separated from singly charged ones. Triply charged ions have ^DT^CCS_N2_ values ranging from 576 to 593 Å^2^, while doubly charged ions range from 538 to 557 Å^2^. The higher ^DT^CCS_N2_ values for triply charged ions compared to doubly charged ones are likely due to charge repulsion effects, which lead to more extended structures. In higher charge states, Coulomb repulsions can dominate intramolecular interactions, causing the structure to unfold to minimize the repulsions [32, 33]. As expected, the loss of the A moiety results in ions with smaller ^DT^CCS _N2_ values, ranging from 497 to 512 Å^2^.

## Conclusions

This study presents, for the first time, the ion mobility behavior of PLTX_std_ and PLTX analogues (OVTX-a and OVTX-b) using the three most frequently used ion mobility instruments (DTIMS, TWIMS, TIMS) for studying small organic molecules and metabolites by LC-IM-HRMS.

The ion mobility results, combined with computational simulations, suggest a "folded" configuration for the chemical structure of PLTX analogues, potentially stabilized by hydrogen bonds involving oxygens and hydroxyl groups located at inner sites of the folded molecule. Additionally, adduct formation such as [M+H+2Na]^3+^ and [M+H+Ca]^3+^ could occur through coordination of 2Na^+^ or Ca^2+^ at inner sites of the folded structure without significantly affecting the total volume and, consequently, the corresponding CCS value. Conversely, the loss of water molecules may involve hydroxyl groups located at the peripheral sites of the folded molecule, as observed in TIMS.

A list of 102 CCS values has been reported, indicating that these toxins exhibit ^DT^CCS_N2_ values ranging from 479 to 593 Å^2^. These values can serve as additional parameters for identifying this family of compounds in complex samples, enhancing sensitivity and selectivity by reducing the background noise in chromatograms (UHPLC-IMS-HRMS) from complex sample extracts. Furthermore, the ^DT^CCS_N2_ values reported in this work can be used as reference values in databases for screening these toxins. The comparison of ^TW^CCS_N2_ values with ^DT^CCS_N2_ values suggests that PLTX_std_ should be used in secondary calibration processes required by TWIMS, TIMS, and cyclic IMS systems to obtain more reliable CCS values for PLTX analogues with these instruments.

## Supplementary Information

Below is the link to the electronic supplementary material.

Supplementary Material 1 (PDF 1.69 MB)

## Data Availability

All data generated or analyzed during this study are included in this article and its supplementary materials.
