# Supplementary material for: Ion mobility–mass spectrometry of palytoxin-like compounds produced by Ostreopsis cf. ovata
Source: Anal Bioanal Chem. 2025 Oct 16;417(29):6665–77. doi: 10.1007/s00216-025-06158-7 (PMC12641035; doi:10.1007/s00216-025-06158-7)
Supplement: Supplementary file 1 — Supplementary Material 1 (PDF 1.69 MB) [file 216_2025_6158_MOESM1_ESM.pdf]

SUPPLEMENTARY MATERIAL

Ion Mobility – Mass Spectrometry of palytoxin-like compounds produced by *Ostreopsis* cf. *ovata*

Noemí Inmaculada Medina-Pérez[1,2] , M. Nuria Peralta-Moreno[3] , Jaime Rubio-Martinez[3] , Leïla Bechtella[4,5], Lukasz Polewski[4,5], Gergo Peter Szekeres[4,5], Elisa Berdalet[2], Encarnación Moyano*[1,6], Kevin Pagel[4,5]

[1] *Department of Chemical Engineering and Analytical Chemistry, University of Barcelona, Barcelona, Spain*

[2] *Department of Marine Biology and Oceanography, Institute of Marine Sciences (ICM-CSIC), Barcelona, Spain*

[3] *Department of Materials Science and Physical Chemistry, University of Barcelona and the Institut de Recerca en Química Teòrica i Computacional (IQTCUB), Barcelona, Spain*

[4] *Department of Molecular Physics, Fritz Haber Institute of the Max Planck Society, Berlin, Germany*

[5] *Department of Chemistry and Biochemistry, Freie Universität Berlin, Berlin, Germany*

[6] *Water Research Institute (IdRA), University of Barcelona, Barcelona, Spain*

Table S1. List of product ions and their corresponding precursor ions. The table also details the applied collision energies, accurate mass measurements, and the IMS system used for CCS measurements.

Table S2. Calculated CCSs values for PLTXstd, OVTX-a, and OVTX-b, measured and estimated using DTIMS and TWIMS. *Refer to Table S1 for product ions.

Table S3. Key hydrogen bonds stablished during the 1 µs simulations for each of the 15 independent replicates of the two OVTX-b adducts. Averaged occupancies were calculated from the complete independent trajectories. Only hydrogen bonds with occupancies greater than 20% of the simulation are included.

Fig. S1. Various perspectives of Na and Ca atoms coordination in the $[M+H+2Na]^{3+}$ and $[M+H+Ca]^{3+}$ adduct ions during the conformational simulation (example of the OVTX-b structure). The colored spheres 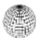 indicate reference positions to enhance visualization of the three-dimensional structure, while yellow dashed lines illustrate Na/Ca coordination.

Figure S2. Hydrogen bonds of the **(a)** $[M+H+2Na]^{3+}$ and **(b)** $[M+H+Ca]^{3+}$ ions observed during the conformational simulation (example of OVTX-b structure). Only hydrogen bonds with occupancies greater than 20% of the simulation are included (see Table S3 for occupancy details).

Figure S3. Radius variation of the $[M+H+Ca]^{3+}$ and $[M+H+2Na]^{3+}$ ions during the conformational simulation (example of the OVTX-b structure).

Figure S4. Hydroxyls groups in the inner (grey) and outer (blue) part of the clustered structures of **(a)** $[M+H+2Na]^{3+}$ and **(b)** $[M+H+Ca]^{3+}$ ions (example of the OVTX-b structure).

Figure S5. Fragmentation of the $[M+2H+Na-3H_2O]^{3+}$ (blue diamond) at CE 35 V for the different ion mobility peaks observed for the OVTX-a using TIMS.

Figure S6. Correlation between CCS values measured using TWIMS or DTIMS instrument, with Dextran or PLTX standard as calibrants.

Table S1. List of product ions and their corresponding precursor ions. The table also details the applied collision energies, accurate mass measurements, and the IMS system used for CCS measurements.

Toxin	Precursor ion	*m/z* (CE)	IMS	Product ion	*m/z*
**$PLTX_{std}$**	**$[M+H+Ca]^{3+}$**	906.4828 (40 V)	**TWIM**	$[M+H+Ca-H_2O]^{3+}$	900.4793
				$[M+H+Ca-2H_2O]^{3+}$	894.4757
	**$[M+H+2Na]^{3+}$**	908.4884 (32 V)	**TWIM**	$[M+H+2Na-2H_2O]^{3+}$	896.4814
				$[M+H+2Na-3H_2O]^{3+}$	890.4779
				$[M+H+2Na-4H_2O]^{3+}$	884.4743
				$[M+H+2Na-5H_2O]^{3+}$	878.4708
	**$[M+H+Na]^{3+}$**	1351.2380 (40–60 V)	**TWIM**	$(\#1+\#4-2H_2O)^+$ (A side charged)	234.1127
				$[M+H\text{–B moiety}-H_2O]^+$	327.1914
				$(\#20-H_2O)^+$ (B side charged)	726.4059
				$(\#19+H-H_2O)^+$ (B side charged)	786.4270
				$(\#19+H)^+$ (B side charged)	804.4376
				$[M+H+Na-2H_2O]^{3+}$	1333.2275
				$[M+H+Na-3H_2O]^{3+}$	1324.2222
				$[M+H+Na-4H_2O]^{3+}$	1315.2169
				$[M+H+Na-5H_2O]^{3+}$	1306.2116
				$[M+H+Na-6H_2O]^{3+}$	1297.2063
				$[M+H+Na-7H_2O]^{3+}$	1288.2011
				$[M+H+Na-8H_2O]^{3+}$	1279.1958
				$[M+H+Na\text{–Amoiety}-4H_2O]^{2+}$	1143.1195
				$[M+H+Na\text{–Amoiety}-5H_2O]^{2+}$	1134.1143
				$[M+H+Na\text{–Amoiety}-6H_2O]^{2+}$	1125.1090
	**$[M+H+Na]^{3+}$**	1351.2380 (50 V)	**DTIM**	$(\#1+\#4-2H_2O)^+$ (A side charged)	234.1127
				$[M+H\text{–B moiety}-H_2O]^+$	327.1914
				$(\#20-2H_2O)^+$ (B side charged)	708.3954
				$(\#20-H_2O)^+$ (B side charged)	726.4059
				$(\#20)^+$ (B side charged)	744.4165
				$(\#19+H-2H_2O)^+$ (B side charged)	768.4165
				$(\#19+H-H_2O)^+$ (B side charged)	786.4270
				$(\#19+H)^+$ (B side charged)	804.4376
				$[M+H+Na\text{–A moiety}-H_2O]^{2+}$	1170.1354
				$[M+H+Na\text{–A moiety}-2H_2O]^{2+}$	1161.1301
				$[M+H+Na\text{–A moiety}-3H_2O]^{2+}$	1152.1248
				$[M+H+Na\text{–A moiety}-4H_2O]^{2+}$	1143.1195
				$[M+H+Na\text{–A moiety}-5H_2O]^{2+}$	1134.1143
				$[M+H+Na\text{–A moiety}-6H_2O]^{2+}$	1125.1090
				$[M+H+Na\text{–A moiety}-7H_2O]^{2+}$	1116.1037
				$[M+H+Na\text{–A moiety}-8H_2O]^{2+}$	1107.0984
				$[M+H+Na-2H_2O]^{2+}$	1333.2275
				$[M+H+Na-3H_2O]^{2+}$	1324.2222
				$[M+H+Na-4H_2O]^{2+}$	1315.2169
				$[M+H+Na-5H_2O]^{2+}$	1306.2116
				$[M+H+Na-6H_2O]^{2+}$	1297.2063
				$[M+H+Na-7H_2O]^{2+}$	1288.2011
				$[M+H+Na-8H_2O]^{2+}$	1279.1958
				$[M+H+Na-9H_2O]^{2+}$	1270.1905

**OVTX-a**	**$[M+H+Ca]^{3+}$**	895.8195 (40 V)	**TWIM**	$[M+H+Ca-H_2O]^{3+}$	889.8160
				$[M+H+Ca-2H_2O]^{3+}$	883.8125
				$[M+H+Ca-3H_2O]^{3+}$	877.8089
				$[M+H+Ca-4H_2O]^{3+}$	871.8054
	**$[M+H+Ca]^{3+}$**	895.8195 (30 V)	**DTIM**	$[M+H+Ca-H_2O]^{3+}$	889.8160
				$[M+H+Ca-2H_2O]^{3+}$	883.8125
				$[M+H+Ca-3H_2O]^{3+}$	877.8089
	**$[M+H+2Na]^{3+}$**	897.8252 (37 V)	**TWIM**	$[M+H+2Na-H_2O]^{3+}$	891.8216
				$[M+H+2Na-2H_2O]^{3+}$	885.8181
				$[M+H+2Na-3H_2O]^{3+}$	879.8146
				$[M+H+2Na-4H_2O]^{3+}$	873.8111
				$[M+H+2Na-5H_2O]^{3+}$	867.8076
				$[M+2Na-Amoiety-2H_2O]^{2+}$	1156.1262
				$[M+2Na-Amoiety-3H_2O]^{2+}$	1147.1209
	**$[M+H+2Na]^{3+}$**	897.8252 (30 V)	**DTIM**	$(\#16+Ca)^{2+}$ (A side charged)	625.3401
				$(\#16+Ca-2H_2O)^{2+}$ (B side charged)	719.8557
				$(\#16+Ca-H_2O)^{2+}$ (B side charged)	728.8610
				$(\#16+Ca)^{2+}$ (B side charged)	737.8663
	**$[M+H+Na]^{2+}$**	1335.2431 (60 V)	**TWIM**	$(\#1+\#4-2H_2O)^{+}$ (A side charged)	234.1127
				$[M+H-B\ moiety-H_2O]^{+}$	327.1914
				$(\#10+\#12)^{+}$ (A side charged)	447.2762
				$(\#15+Ca)^{2+}$ (A side charged)	588.3217
				$(\#16+Ca)^{2+}$ (A side charged)	625.3401
				$(\#16+Ca-2H_2O)^{2+}$ (B side charged)	719.8557
				$(\#16+Ca-H_2O)^{2+}$ (B side charged)	728.8610
				$(\#16+Ca)^{2+}$ (B side charged)	737.8663
				$(\#19+H)^{+}$ (B side charged)	804.4376
				$(\#19+H-H_2O)^{+}$ (B side charged)	786.4270
				$[M+H+Na-2H_2O]^{3+}$	1317.2326
				$[M+H+Na-3H_2O]^{3+}$	1308.2273
				$[M+H+Na-4H_2O]^{3+}$	1299.2220
				$[M+H+Na-5H_2O]^{3+}$	1290.2167
				$[M+H+Na-6H_2O]^{3+}$	1281.2114
				$[M+H+Na-7H_2O]^{3+}$	1272.2062
				$[M+H+Na-Amoiety\text{-}H_2O]^{2+}$	1154.1405
				$[M+H+Na-Amoiety\text{-}2H_2O]^{2+}$	1145.1352
				$[M+H+Na-Amoiety\text{-}3H_2O]^{2+}$	1136.1299
				$[M+H+Na-Amoiety\text{-}4H_2O]^{2+}$	1127.1246
				$[M+H+Na-Amoiety\text{-}5H_2O]^{2+}$	1118.1193

**OVTX-a**	**$[M+H+Na]^{2+}$**	1335.2431 (45 V)	**DTIM**	$[M+H−B\ moiety−H_2O]^{+}$	327.1914
				$[M+H+Na−A\ moiety]^{2+}$	1163.1458
				$[M+H+Na−A\ moiety-H_2O]^{2+}$	1154.1405
				$[M+H+Na−A\ moiety-2H_2O]^{2+}$	1145.1352
				$[M+H+Na−A\ moiety-3H_2O]^{2+}$	1136.1299
				$[M+H+Na−A\ moiety-4H_2O]^{2+}$	1127.1246
				$[M+H+Na−A\ moiety-5H_2O]^{2+}$	1118.1193
				$[M+H+Na−2H_2O]^{3+}$	1317.2326
				$[M+H+Na−3H_2O]^{3+}$	1308.2273
				$[M+H+Na−4H_2O]^{3+}$	1299.2220
				$[M+H+Na−5H_2O]^{3+}$	1290.2167
				$[M+H+Na−6H_2O]^{3+}$	1281.2114
				$[M+H+Na−7H_2O]^{3+}$	1272.2062
**OVTX-b**	**$[M+H+Na]^{3+}$**	1357.2562 (42 V)	**TWIM**	$[M+H+Na−H_2O]^{2+}$	1348.2510
	**$[M+H+Na]^{3+}$**	1357.2562 (27 V)	**DTIM**	$[M+H+Na−2H_2O]^{2+}$	1339.2457
	**$[M+H+Ca]^{3+}$**	910.4950 (25 V)	**DTIM**	$[M+H+Ca−H_2O]^{3+}$	904.4914

Table S2. Calculated CCSs values for PLTXstd, OVTX-a, and OVTX-b, measured and estimated using DTIMS and TWIMS. *Refer to Table S1 for product ions.

			DTIMS								TWIMS calibrant: dextran		TWIMS calibrant: PLTXstd	
			Helium gas				Nitrogen gas							
			Intraday		Interday		Intraday		Interday		Interday		Interday	
Toxin	Ion	*m/z*	$^{DT}CCS_{He}$ (Å²)	SD	$^{DT}CCS_{He}$ (Å²)	SD	$^{DT}CCS_{N2}$ (Å²)	SD	$^{DT}CCS_{N2}$ (Å²)	SD	$^{TW}CCS_{N2}$ (Å²)	SD	$^{TW}CCS_{N2}$ (Å²)	SD
PLTX	$(\#1+\#4-2H_2O)^+$	234.1127	*95.6	0.1	*95.3	0.4	nd	nd	nd	nd	*180.5	0.1	*170.0	0.3
PLTX	$[M+H-B\ moiety-H_2O]^+$	327.1914	*120.6	0.1	*120.7	0.6	*184.6	0.5	*184.6	2.1	188.6	0.2	181.5	3.3
PLTX	$(\#20-2H_2O)^+$	708.3954	*186.6	1.5	*186.7	0.1	nd	nd	nd	nd	nd	nd	nd	nd
PLTX	$(\#20-H_2O)^+$	726.4059	*186.3	0.7	*186.9	1.2	*275.5	0.6	*275.0	3.4	*276.0	2.1	*269.5	2.7
PLTX	$(\#20)^+$	744.4165	*187.9	0.5	*186.8	0.8	nd	nd	nd	nd	nd	nd	nd	nd
PLTX	$(\#19+H-2H_2O)^+$	768.4165	*193.9	1.4	*193.0	1.0	*263.5	0.5	*263.3	2.4	nd	nd	nd	nd
PLTX	$(\#19+H-H_2O)^+$	786.4270	*192.6	0.2	*192.2	0.7	nd	nd	nd	nd	*273.5	3.2	*259.3	0.3
PLTX	$(\#19+H)^+$	804.4376	*191.6	0.4	*191.1	0.3	nd	nd	nd	nd	*270.0	1.0	*258.5	0.3
PLTX	$[M+3H-7H_2O]^{3+}$	851.8090	nd	nd	nd	nd	nd	nd	nd	nd	644.9	0.9	612.5	1.1
PLTX	$[M+H+Mg-3H_2O]^{3+}$	883.1464	nd	nd	nd	nd	nd	nd	nd	nd	647.3	0.3	595.5	0.1
PLTX	$*[M+H+2Na-5H_2O]^{3+}$	878.4708	nd	nd	nd	nd	nd	nd	nd	nd	*620.9	1.8	*588.9	2.0
PLTX	$*[M+H+2Na-4H_2O]^{3+}$	884.4743	nd	nd	nd	nd	nd	nd	nd	nd	*623.6	0.5	*591.6	0.3
PLTX	$*[M+H+2Na-3H_2O]^{3+}$	890.4779	462.5	1.3	462.5	2.5	576.4	2.9	581.9	3.9	*622.6	0.7	*590.6	0.4
PLTX	$*[M+H+2Na-2H_2O]^{3+}$	896.4814	nd	nd	nd	nd	nd	nd	nd	nd	*622.9	0.2	*590.9	0.5
PLTX	$[M+H+2Na-H_2O]^{3+}$	902.4849	nd	nd	nd	nd	577.3	1.9	582.7	4.6	624.2	0.4	592.2	0.1
PLTX	$[M+H+2Na]^{3+}$	908.4884	440.1	1.6	441.7	3.4	577.9	2.0	583.5	4.2	623.6	0.2	591.6	0.1

			DTIMS								TWIMS calibrant: dextran		TWIMS calibrant: PLTXstd	
			Helium gas				Nitrogen gas							
			Intraday		Interday		Intraday		Interday		Interday		Interday	
Toxin	Ion	*m/z*	$^{DT}CCS_{He}$ (Å²)	SD	$^{DT}CCS_{He}$ (Å²)	SD	$^{DT}CCS_{N2}$ (Å²)	SD	$^{DT}CCS_{N2}$ (Å²)	SD	$^{TW}CCS_{N2}$ (Å²)	SD	$^{TW}CCS_{N2}$ (Å²)	SD
PLTX	$[M+H+Ca-4H_2O]_{3+}$	882.4687	469.9	1.0	469.2	0.7	579.2	1.6	580.2	1.1	nd	nd	nd	nd
PLTX	*$[M+H+Ca-3H_2O]^{3+}$	888.4722	467.9	1.4	466.9	0.9	579.1	1.4	585.8	4.8	nd	nd	nd	nd
PLTX	*$[M+H+Ca-2H_2O]^{3+}$	894.4757	461.3	1.5	459.9	2.1	581.5	1.4	586.5	3.9	*629.3	0.2	*597.2	0.4
PLTX	*$[M+H+Ca-H_2O]^{3+}$	900.4793	444.1	1.8	445.0	2.9	581.6	1.2	586.7	3.9	*630.4	0.4	*598.3	0.6
PLTX	$[M+H+Ca]^{3+}$	906.4828	442.0	1.6	443.4	3.5	581.0	1.6	586.3	4.1	623.8	0.1	591.8	0.3
PLTX	$[M+H+Fe]^{3+}$	911.1418	443.2	1.5	442.3	0.9	582.1	1.8	587.1	4.0	623.5	0.1	591.6	0.3
PLTX	$[M+H+Na-A\ moiety-8H_2O]^{2+}$	1107.0984	*389.3	0.7	*390.0	1.0	*496.7	2.9	*497.6	6.6	nd	nd	nd	nd
PLTX	$[M+H+Na-A\ moiety-7H_2O]^{2+}$	1116.1037	*392.1	0.1	*391.7	0.7	*501.4	0.6	*498.7	2.7	nd	nd	nd	nd
PLTX	$[M+H+Na-A\ moiety-6H_2O]^{2+}$	1125.1090	*392.4	0.8	*392.0	0.7	*499.4	0.9	*501.3	1.9	*521.5	0.8	*498.3	0.8
PLTX	$[M+H+Na-A\ moiety-5H_2O]^{2+}$	1134.1143	*393.1	0.1	*393.4	1.1	*502.9	1.6	*503.2	0.3	*522.5	0.5	*499.3	0.5
PLTX	$[M+H+Na-A\ moiety-4H_2O]^{2+}$	1143.1195	*394.4	0.6	*394.2	0.7	*503.1	0.9	*505.2	1.6	*524.4	0.2	*501.2	0.3
PLTX	$[M+H+Na-A\ moiety-3H_2O]^{2+}$	1152.1248	*394.6	0.2	*394.7	0.8	*504.8	0.6	*505.3	1.1	525.0	0.8	501.8	0.8
PLTX	$[M+H+Na-A\ moiety-2H_2O]^{2+}$	1161.1301	*397.0	0.4	*396.5	0.8	*507.5	1.9	*508.7	1.3	527.7	0.4	504.5	0.4
PLTX	$[M+H+Na-A\ moiety-H_2O]^{2+}$	1170.1354	*396.6	0.1	*396.8	0.4	*508.8	2.6	*507.6	1.6	528.8	0.4	505.5	0.5
PLTX	$[M+H+Na-9H_2O]^{2+}$	1270.1905	*429.3	0.1	*429.3	0.7	*537.9	1.0	*538.1	0.2	nd	nd	nd	nd
PLTX	$[M+H+Na-8H_2O]^{2+}$	1279.1958	*429.7	0.2	*429.9	0.5	*541.6	1.0	*541.9	0.4	*566.9	0.8	*542.9	0.8
PLTX	$[M+H+Na-7H_2O]^{2+}$	1288.2011	*430.5	0.1	*430.4	0.9	*541.0	0.4	*542.4	1.1	*566.6	0.3	*542.7	0.4
PLTX	$[M+H+Na-6H_2O]^{2+}$	1297.2063	*430.8	0.2	*430.9	0.6	*542.2	0.0	*543.2	0.9	*566.9	0.1	*543.0	0.5
PLTX	$[M+H+Na-5H_2O]^{2+}$	1306.2116	*432.3	0.3	*432.2	0.7	*542.7	1.2	*544.6	1.6	*568.3	0.4	*544.4	0.4

			DTIMS								TWIMS calibrant: dextran		TWIMS calibrant: PLTXstd	
			Helium gas				Nitrogen gas							
			Intraday		Interday		Intraday		Interday		Interday		Interday	
**Toxin**	**Ion**	***m/z***	$^{DT}CCS_{He}$ (Å^2)	SD	$^{DT}CCS_{He}$ (Å^2)	SD	$^{DT}CCS_{N2}$ (Å^2)	SD	$^{DT}CCS_{N2}$ (Å^2)	SD	$^{TW}CCS_{N2}$ (Å^2)	SD	$^{TW}CCS_{N2}$ (Å^2)	SD
**PLTX**	$[M+H+Na-4H_2O]^{2+}$	1315.2169	*432.8	0.1	*432.7	0.5	*543.6	0.7	*546.1	1.8	*570.1	0.3	*546.1	0.3
**PLTX**	$[M+H+Na-3H_2O]^{2+}$	1324.2222	*433.2	0.1	*433.2	0.6	*545.9	0.5	*547.3	1.0	*571.2	0.5	*547.2	0.4
**PLTX**	$[M+H+Na-2H_2O]^{2+}$	1333.2275	*433.5	0.1	*433.6	0.6	*546.3	0.5	*547.4	1.1	*572.6	0.1	*548.5	0.1
**PLTX**	$[M+H+Na-H_2O]^{2+}$	1342.2328	431.0	0.4	432.6	1.6	546.7	0.8	550.4	3.1	572.6	1.2	548.6	1.2
**PLTX**	$[M+H+Na]^{2+}$	1351.2380	432.6	0.9	431.6	0.7	551.7	5.4	548.8	2.5	569.7	0.3	545.7	0.3
**PLTX**	$[M+2H-4H_2O]^{2+}$	1304.2259	nd	nd	nd	nd	nd	nd	nd	nd	564.4	0.6	540.6	0.5
**PLTX**	$[M+2H-3H_2O]^{2+}$	1313.2312	nd	nd	nd	nd	nd	nd	nd	nd	566.0	0.7	542.1	0.7
**PLTX**	$[M+2H-2H_2O]^{2+}$	1322.2365	nd	nd	nd	nd	nd	nd	nd	nd	565.9	0.8	542.0	0.7
**PLTX**	$[M+2H-H_2O]^{2+}$	1331.2418	nd	nd	nd	nd	nd	nd	nd	nd	567.0	1.0	542.9	2.2
**PLTX**	$[M+H+K]^{2+}$	1359.2250	433.4	0.5	435.3	1.7	551.5	1.6	555.2	4.3	572.6	0.4	548.6	0.4
**PLTX**	$[M+2Na]^{2+}$	1362.2290	437.0	0.6	439.2	1.6	553.4	2.6	556.5	2.2	573.1	0.3	549.1	0.2
**OVTX-a**	$(\#1+\#4-2H_2O)^{+}$	234.1127	na	na	na	na	nd	nd	nd	nd	*172.0	0.1	*161.5	0.2
**OVTX-a**	$[M+H-B\ moiety-H_2O]^{+}$	327.1914	na	na	na	na	*181.7	0.5	*180.7	1.1	*185.9	0.1	*182.4	0.2
**OVTX-a**	$(\#10+\#12)^{+}$	447.2762	na	na	na	na	nd	nd	nd	nd	*215.4	0.2	*204.4	1.0
**OVTX-a**	$(\#15+Ca)^{2+}$	588.3217	na	na	na	na	nd	nd	nd	nd	*390.8	0.2	*369.9	0.4
**OVTX-a**	$(\#16+Ca)^{2+}$	625.3401	na	na	na	na	*378.5	2.3	*377.0	1.5	*401.0	0.3	*379.8	0.5
**OVTX-a**	$(\#16+Ca-2H_2O)^{2+}$	719.8557	na	na	na	na	*392.9	0.4	*392.6	0.2	*412.7	0.2	*391.2	1.1
**OVTX-a**	$(\#16+Ca-H_2O)^{2+}$	728.8610	na	na	na	na	*393.6	0.8	*394.5	0.9	*414.4	0.3	*393.1	0.5
**OVTX-a**	$(\#16+Ca)^{2+}$	737.8663	na	na	na	na	*393.1	1.3	*392.7	0.4	*413.4	0.2	*392.1	0.1

Toxin	Ion	*m/z*	DTIMS Helium gas Intraday $^{DT}CCS_{He}$ (Å²)	SD	DTIMS Helium gas Interday $^{DT}CCS_{He}$ (Å²)	SD	DTIMS Nitrogen gas Intraday $^{DT}CCS_{N2}$ (Å²)	SD	DTIMS Nitrogen gas Interday $^{DT}CCS_{N2}$ (Å²)	SD	TWIMS calibrant: dextran, Nitrogen gas Interday $^{TW}CCS_{N2}$ (Å²)	SD	TWIMS calibrant: PLTXstd, Nitrogen gas Interday $^{TW}CCS_{N2}$ (Å²)	SD
**OVTX-a**	**$(\#19+H-H_2O)^+$**	786.4270	na	na	na	na	nd	nd	nd	nd	*270.0	0.6	*258.2	0.6
**OVTX-a**	**$(\#19+H)^+$**	804.4376	na	na	na	na	nd	nd	nd	nd	*270.5	0.1	*258.6	0.1
**OVTX-a**	**$[M+H+Mg-4H_2O]^{3+}$**	866.4796	na	na	na	na	nd	nd	nd	nd	627.8	0.2	601.1	1.7
**OVTX-a**	**$[M+H+Mg-3H_2O]^{3+}$**	872.4831	na	na	na	na	nd	nd	nd	nd	627.1	0.2	594.7	1.8
**OVTX-a**	**$[M+H+Mg-2H_2O]^{3+}$**	878.4866	na	na	na	na	nd	nd	nd	nd	627.0	1.3	594.7	1.0
**OVTX-a**	**$[M+H+Mg-H_2O]^{3+}$**	884.4901	na	na	na	na	nd	nd	nd	nd	627.6	0.7	595.3	1.5
**OVTX-a**	**$[M+H+Mg]^{3+}$**	890.4937	na	na	na	na	nd	nd	nd	nd	626.2	0.8	594.0	1.3
**OVTX-a**	**$[M+H+Ca-4H_2O]^{3+}$**	871.8054	na	na	na	na	nd	nd	nd	nd	*633.9	1.3	*601.5	1.6
**OVTX-a**	**$[M+H+Ca-3H_2O]^{3+}$**	877.8089	na	na	na	na	*592.1	1.1	591.7	0.4	*631.4	1.5	*599.3	1.2
**OVTX-a**	**$[M+H+Ca-2H_2O]^{3+}$**	883.8125	na	na	na	na	*589.3	1.0	589.9	0.6	*630.1	0.9	*598.0	0.6
**OVTX-a**	**$[M+H+Ca-H_2O]^{3+}$**	889.8160	na	na	na	na	*587.0	0.2	587.7	0.7	*628.5	0.1	*596.4	0.2
**OVTX-a**	**$[M+H+Ca]^{3+}$**	895.8195	na	na	na	na	*584.8	0.2	586.1	1.3	624.4	0.2	592.4	0.5
**OVTX-a**	**$[M+H+2Na-5H_2O]^{3+}$**	867.8076	na	na	na	na	nd	nd	nd	nd	*634.2	0.8	*602.0	0.6
**OVTX-a**	**$[M+H+2Na-4H_2O]^{3+}$**	873.8111	na	na	na	na	nd	nd	nd	nd	*629.7	0.1	*597.6	0.2
**OVTX-a**	**$[M+H+2Na-3H_2O]^{3+}$**	879.8146	na	na	na	na	nd	nd	nd	nd	*626.3	0.8	*594.3	0.5
**OVTX-a**	**$[M+H+2Na-2H_2O]^{3+}$**	885.8181	na	na	na	na	nd	nd	nd	nd	*626.0	0.4	*594.0	0.1
**OVTX-a**	**$[M+H+2Na-H_2O]^{3+}$**	891.8216	na	na	na	na	582.5	1.3	583.6	1.1	*628.4	0.3	*595.4	0.1
**OVTX-a**	**$[M+H+2Na]^{3+}$**	897.8252	na	na	na	na	582.5	1.0	582.5	1.6	624.1	1.6	592.3	1.3
**OVTX-a**	**$[M+2Na-A\ moiety-3H_2O]^{2+}$**	1147.1209	na	na	na	na	nd	nd	nd	nd	*782.5	0.3	*747.8	0.2

			DTIMS								TWIMS calibrant: dextran		TWIMS calibrant: PLTXstd	
			Helium gas				Nitrogen gas							
			Intraday		Interday		Intraday		Interday		Interday		Interday	
**Toxin**	**Ion**	***m/z***	$^{DT}CCS_{He}$ (Å^2)	SD	$^{DT}CCS_{He}$ (Å^2)	SD	$^{DT}CCS_{N2}$ (Å^2)	SD	$^{DT}CCS_{N2}$ (Å^2)	SD	$^{TW}CCS_{N2}$ (Å^2)	SD	$^{TW}CCS_{N2}$ (Å^2)	SD
**OVTX-a**	**[M+2Na−A moiety−2H$_2$O]$^{2+}$**	1156.1262	na	na	na	na	nd	nd	nd	nd	*786.0	0.9	*751.2	1.0
**OVTX-a**	**[M+H+Na−A moiety−5H$_2$O]$^{2+}$**	1118.1193	na	na	na	na	*504.3	0.7	*503.8	0.5	*521.2	0.2	*498.1	0.2
**OVTX-a**	**[M+H+Na−A moiety-4H$_2$O]$^{2+}$**	1127.1246	na	na	na	na	*503.3	0.2	*504.2	0.9	*522.0	0.2	*498.9	0.2
**OVTX-a**	**[M+H+Na−A moiety−3H$_2$O]$^{2+}$**	1136.1299	na	na	na	na	*505.8	0.4	*506.7	1.0	*525.1	0.0	*501.9	0.1
**OVTX-a**	**[M+H+Na−A moiety−2H$_2$O]$^{2+}$**	1145.1352	na	na	na	na	*507.0	1.2	*507.3	0.3	*525.2	0.4	*502.0	0.4
**OVTX-a**	**[M+H+Na-A moiety−H$_2$O]$^{2+}$**	1154.1405	na	na	na	na	*507.5	0.1	*508.5	1.0	*527.4	0.3	*504.1	0.3
**OVTX-a**	**[M+H+Na−A moiety]$^{2+}$**	1163.1458	na	na	na	na	*511.5	1.4	*513.3	1.8	nd	nd	nd	nd
**OVTX-a**	**[M+2H−5H$_2$O]$^{2+}$**	1279.2257	na	na	na	na	546.7	0.9	547.9	1.2	nd	nd	nd	nd
**OVTX-a**	**[M+2H−4H$_2$O]$^{2+}$**	1288.2310	na	na	na	na	548.1	1.0	549.1	1.0	563.2	0.3	539.3	0.3
**OVTX-a**	**[M+2H−3H$_2$O]$^{2+}$**	1297.2363	na	na	na	na	551.0	0.8	551.3	0.3	571.3	0.2	547.3	0.1
**OVTX-a**	**[M+2H−2H$_2$O]$^{2+}$**	1306.2416	na	na	na	na	nd	nd	nd	nd	572.6	0.9	548.6	1.0
**OVTX-a**	**[M+H+Na−7H$_2$O]$^{2+}$**	1272.2062	na	na	na	na	*543.3	0.4	*543.4	0.1	*564.8	0.9	*540.9	0.9
**OVTX-a**	**[M+H+Na−6H$_2$O]$^{2+}$**	1281.2114	na	na	na	na	*542.0	0.7	*543.1	1.1	*566.7	1.2	*542.8	0.1
**OVTX-a**	**[M+H+Na−5H$_2$O]$^{2+}$**	1290.2167	na	na	na	na	*544.2	0.9	*545.0	0.8	*567.6	0.5	*543.6	0.5
**OVTX-a**	**[M+H+Na−4H$_2$O]$^{2+}$**	1299.2220	na	na	na	na	*544.3	0.3	*544.6	0.3	*568.3	0.5	*544.3	0.4
**OVTX-a**	**[M+H+Na−3H$_2$O]$^{2+}$**	1308.2273	na	na	na	na	*545.9	0.8	*546.3	0.4	*569.7	1.0	*545.7	0.1
**OVTX-a**	**[M+H+Na−2H$_2$O]$^{2+}$**	1317.2326	na	na	na	na	*546.8	0.1	*547.4	0.5	*571.2	0.1	547.2	0.1
**OVTX-a**	**[M+H+Na−H$_2$O]$^{2+}$**	1326.2378	na	na	na	na	548.2	0.2	548.5	0.3	571.9	0.1	547.9	0.2
**OVTX-a**	**[M+H+Na]$^{2+}$**	1335.2431	na	na	na	na	554.3	0.5	554.2	0.1	568.6	0.6	544.6	0.5

			DTIMS								TWIMS calibrant: dextran		TWIMS calibrant: PLTXstd	
			Helium gas				Nitrogen gas							
			Intraday		Interday		Intraday		Interday		Interday		Interday	
**Toxin**	**Ion**	***m/z***	$^{DT}CCS_{He}$ (Å²)	**SD**	$^{DT}CCS_{He}$ (Å²)	**SD**	$^{DT}CCS_{N2}$ (Å²)	**SD**	$^{DT}CCS_{N2}$ (Å²)	**SD**	$^{TW}CCS_{N2}$ (Å²)	**SD**	$^{TW}CCS_{N2}$ (Å²)	**SD**
**OVTX-a**	$[M+H+K]^{2+}$	1343.2301	na	na	na	na	557.0	1.6	557.1	0.1	571.7	0.1	547.6	0.8
**OVTX-a**	$[M+2Na]^{2+}$	1346.2341	na	na	na	na	557.2	0.7	557.1	0.1	570.8	0.9	546.9	0.9
**OVTX-b**	$[M+H+Ca-H_2O]^{3+}$	904.4910	na	na	na	na	*592.9	2.3	*593.5	0.6	nd	nd	nd	nd
**OVTX-b**	$[M+H+Ca]^{3+}$	910.4950	na	na	na	na	590.8	0.2	593.2	2.4	625.4	0.8	593.4	0.5
**OVTX-b**	$[M+2H-4H_2O]^{2+}$	1310.2441	na	na	na	na	nd	nd	nd	nd	571.0	0.2	547.0	0.2
**OVTX-b**	$[M+2H-3H_2O]^{2+}$	1319.2494	na	na	na	na	nd	nd	nd	nd	573.9	0.5	549.9	0.5
**OVTX-b**	$[M+H+Na-2H_2O]^{2+}$	1339.2457	na	na	na	na	*548.9	0.6	*550.4	1.5	nd	nd	nd	nd
**OVTX-b**	$[M+H+Na-H_2O]^{2+}$	1348.2510	na	na	na	na	549.8	0.1	549.8	3.2	*576.1	0.6	*552.1	0.6
**OVTX-b**	$[M+H+Na]^{2+}$	1357.2562	na	na	na	na	549.6	0.7	556.6	7.0	574.0	0.2	549.9	0.2
**OVTX-b**	$[M+H+K]^{2+}$	1365.2432	na	na	na	na	552.7	0.2	560.1	7.4	nd	nd	nd	nd

nd: non detected
na: non analyzed

Table S3. Key hydrogen bonds stablished during the 1 μs simulations for each of the 15 independent replicates of the two OVTX-b adducts. Averaged occupancies were calculated from the complete independent trajecto-ries. Only hydrogen bonds with occupancies greater than 20% of the simulation are included.

System	Acceptor	Donor	Occupancy (%)
$[M+H+2Na]^{3+}$	O33	O32-H166	59.0
	O46	O47-H214	54.3
	O4	O25-H159	45.6
	O5	O15-H149	42.2
	O30	O29-H163	41.1
	O31	O32-H166	36.1
	O12	O14-H148	30.9
	O32	O31-H165	30.5
	O32	O33-H167	29.6
	O42	O43-H210	28.5
	O5	O11-H145	24.7
	O21	O22-H156	22.6
	O3	O29-H163	22.2
	O35	O42-H209	22.1
$[M+H+Ca]^{3+}$	O33	O32-H166	52.0
	O46	O47-H214	39.8
	O30	O29-H163	36.0
	O4	O25-H159	35.2
	O12	O14-H148	33.9
	O16	O17-H151	31.4
	O6	O10-H144	30.0
	O31	O32-H166	29.5
	O49	O47-H214	29.3
	O20	O18-H152	29.2
	O5	O15-H149	27.2
	O17	O15-H149	25.2
	O4	O21-H155	24.6
	O34	O28-H162	20.0

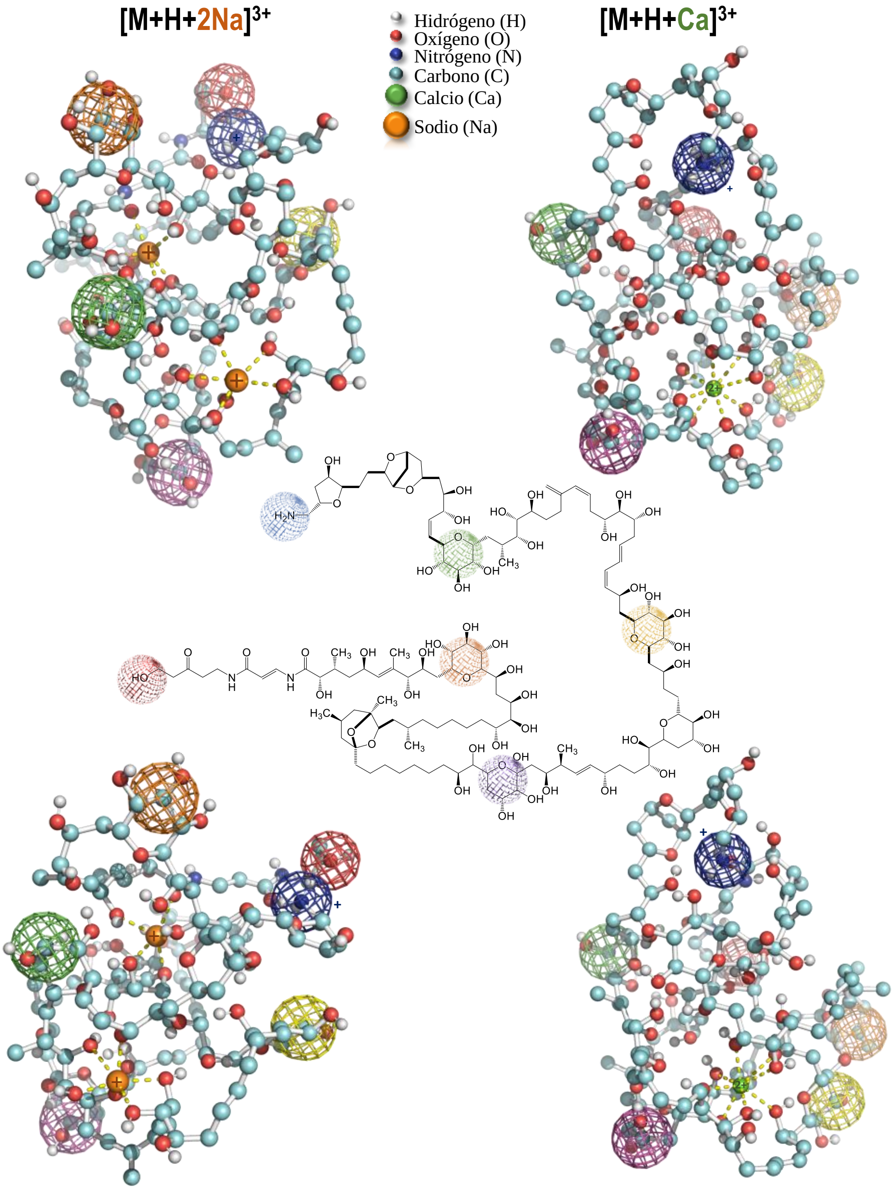

Fig. S1. Various perspectives of Na and Ca atoms coordination in the $[M+H+2Na]^{3+}$ and $[M+H+Ca]^{3+}$ adduct ions during the conformational simulation (example of the OVTX-b structure). The colored spheres 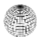 indicate reference positions to enhance visualization of the three-dimensional structure, while yellow dashed lines illustrate Na/Ca coordination.

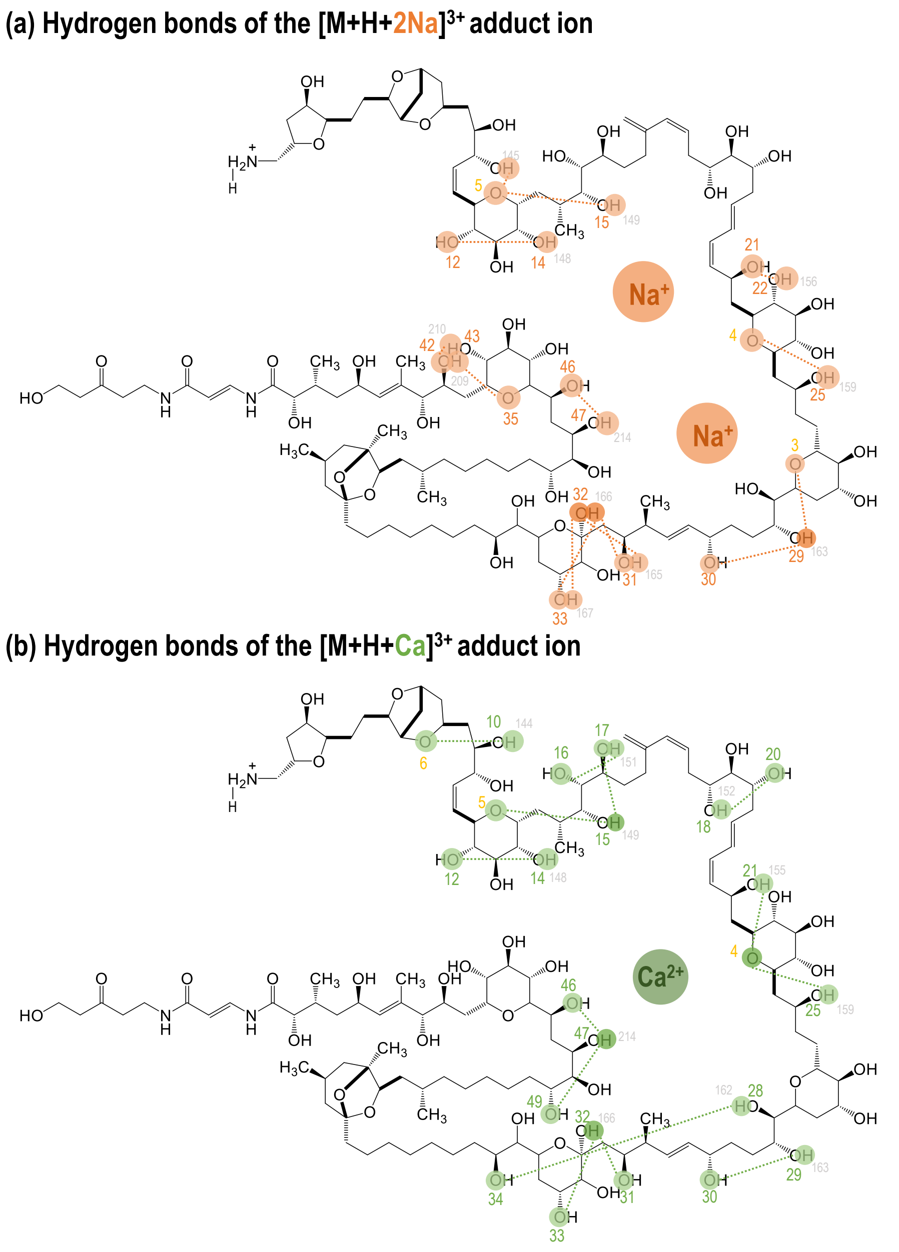

Fig. S2. Hydrogen bonds of the (a) $[M+H+2Na]^{3+}$ and (b) $[M+H+Ca]^{3+}$ ions observed during the conformational simulation (example of OVTX-b structure). Only hydrogen bonds with occupancies greater than 20% of the simulation are included (see Table S3 for occupancy details).

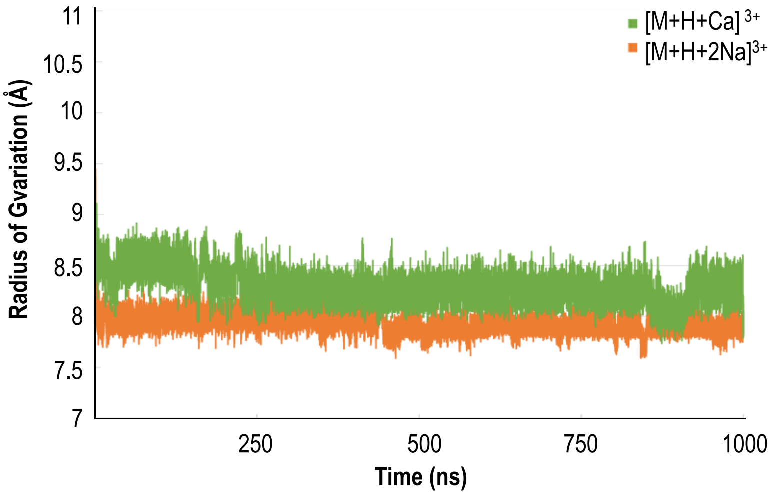

Fig. S3. Radius variation of the [M+H+Ca]3+ and [M+H+2Na]3+ ions during the conformational simulation, illustrated using the OVTX-b structure as an example. The plot shows how the radius of each ion evolves over time, reflecting differences in structural flexibility and compactness.

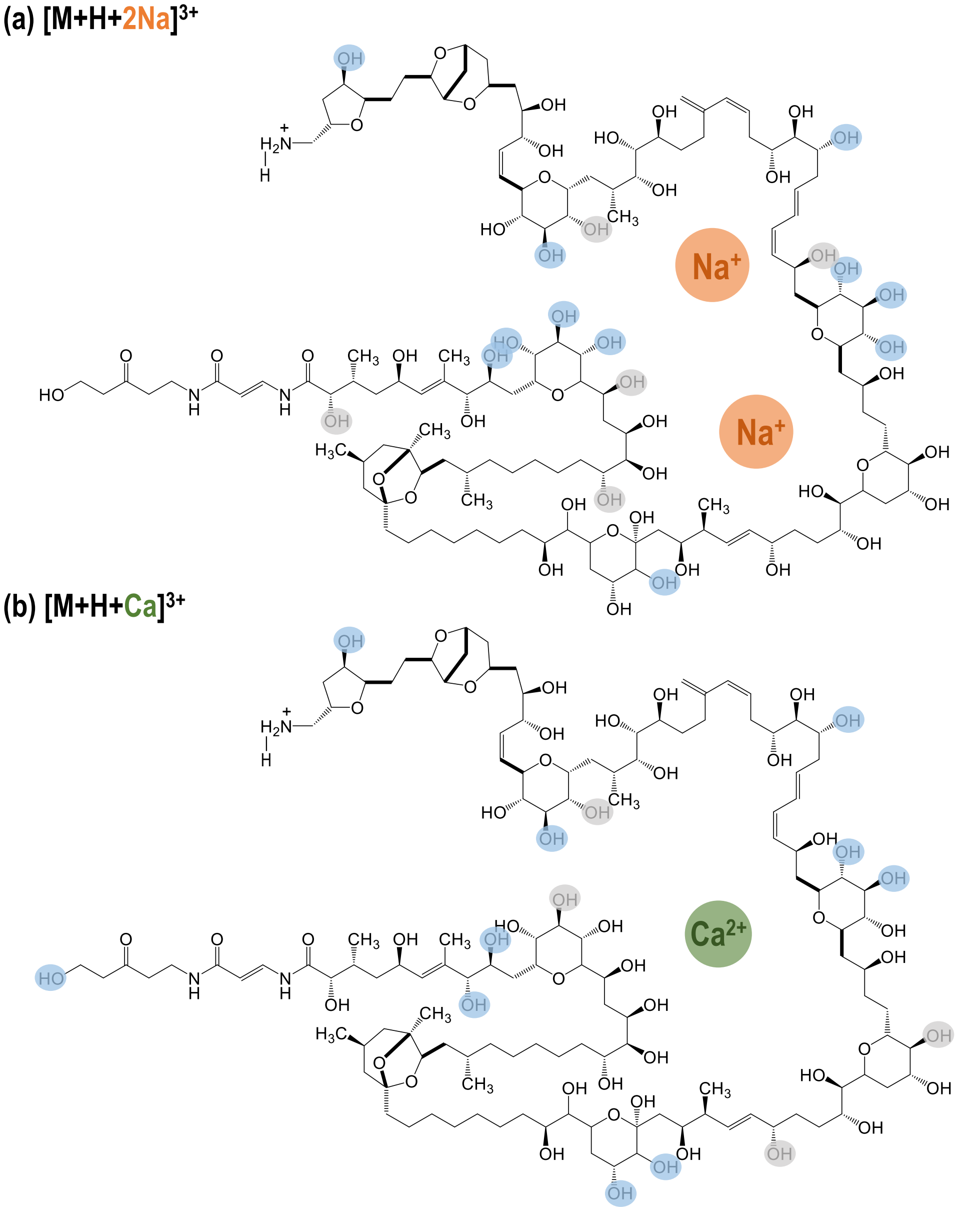

Fig. S4. Hydroxyls groups in the inner (grey) and outer (blue) part of the clustered structures of **(a)** $[M+H+2Na]^{3+}$ and **(b)** $[M+H+Ca]^{3+}$ ions (example of the OVTX-b structure).

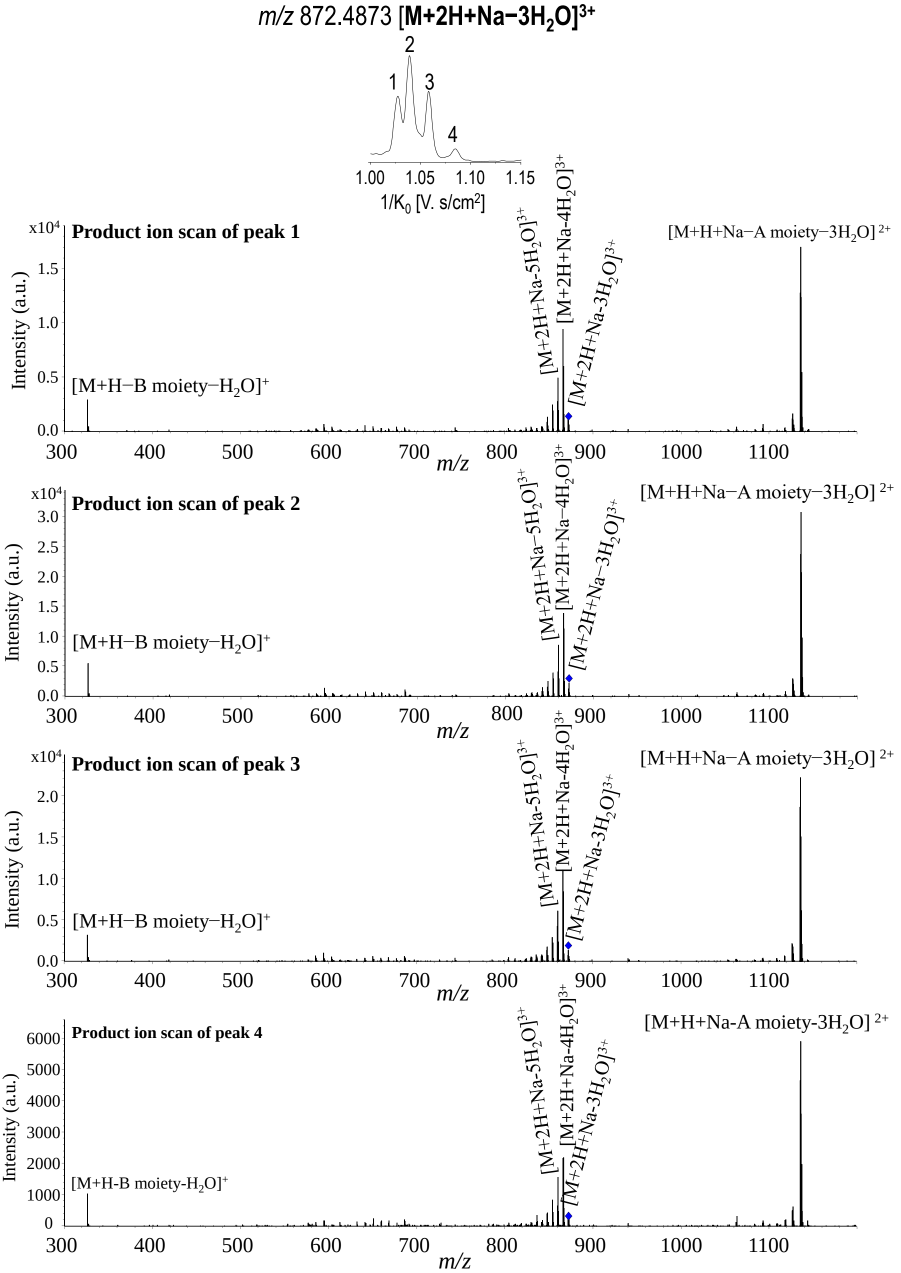

Figure S5. Fragmentation of the [M+2H+Na−3H$_2$O]$^{3+}$ (blue diamond) at CE 35 V for the different ion mobility peaks observed for the OVTX-a using TIMS.

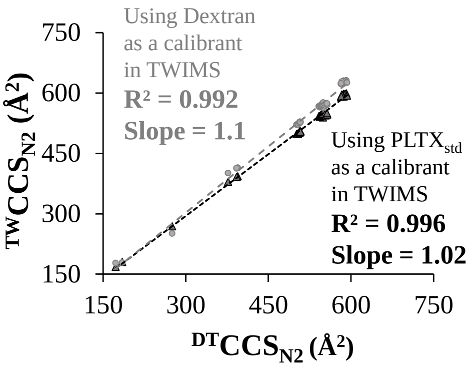

Figure S6. Correlation between CCS values measured using TWIMS or DTIMS instrument, with Dextran or PLTX standard as calibrants.
